# PGC1α promotes cholangiocarcinoma metastasis by upregulating PDHA1 and MPC1 expression to reverse the Warburg effect

**DOI:** 10.1038/s41419-018-0494-0

**Published:** 2018-04-27

**Authors:** Dan Li, Chaoqun Wang, Panfei Ma, Qingan Yu, Mingqi Gu, Liqian Dong, Wenjing Jiang, Shangha Pan, Changming Xie, Jihua Han, Yaliang Lan, Jing Sun, Ping Sheng, Kunpeng Liu, Yaohua Wu, Lianxin Liu, Yong Ma, Hongchi Jiang

**Affiliations:** 1 Department of Hepatic Surgery, The First Affiliated Hospital of Harbin Medical University, Key Laboratory of Hepatosplenic Surgery, Ministry of Education, Harbin, Heilongjiang Province China; 2 Key Laboratory of Hepatosplenic Surgery, Ministry of Education, The First Affiliated Hospital of Harbin Medical University, Harbin, Heilongjiang Province China

## Abstract

PGC1α acts as a central regulator of mitochondrial metabolism, whose role in cancer progression has been highlighted but remains largely undefined. Especially, it is completely unknown about the effect of PGC1α on cholangiocarcinoma (CCA). Here we showed that PGC1α overexpression had no impact on CCA growth despite the decreased expression of PGC1α in CCA compared with adjacent normal tissue. Instead, PGC1α overexpression-promoted CCA metastasis both *in vitro* and *in vivo*. Mechanistically, for the first time, we illuminated that PGC1α reversed the Warburg effect by upregulating the expression of pyruvate dehydrogenase E1 alpha 1 subunit and mitochondrial pyruvate carrier 1 to increase pyruvate flux into the mitochondria for oxidation, whereas simultaneously promoting mitochondrial biogenesis and fusion to mediate the metabolic switch to oxidative phosphorylation. On the one hand, enhanced mitochondrial oxidation metabolism correlated with elevated reactive oxygen species (ROS) production; on the other hand, increased PGC1α expression upregulated the expression levels of mRNA for several ROS-detoxifying enzymes. To this end, the ROS levels, which were elevated but below a critical threshold, did not inhibit CCA cells proliferation. And the moderately increased ROS facilitated metastatic dissemination of CCA cells, which can be abrogated by antioxidants. Our study suggests the potential utility of developing the PGC1α-targeted therapies or blocking PGC1α signaling axis for inhibiting CCA metastasis.

## Introduction

Recently, the overall incidence of cholangiocarcinoma (CCA) appears to have increased^1,2^, and cumulative CCA mortality has risen by 39% because of increased disease incidence^3^. The difficulty in diagnosing CCA in the early stage, the low rate of surgical resection and its therapeutic resistance all contribute to its unfavorable prognosis. Therefore, there is an urgent need to understand the molecular mechanism underlying CCA progression.

Currently, metabolic reprogramming is now firmly established as “Achilles’ heel” of cancer cells^4^. Tumors are even regarded as a metabolic disease^5^. The best characterized energy metabolism modification in cancer cells is the Warburg effect, wherein tumor cells oxidize a reduced fraction of the pyruvate generated from glycolysis. It is the long-term correlation of cancer metabolism with aerobic glycolysis that strikingly belies the crucial role of mitochondria in cancer cells^6^. Contrary to Otto Warburg’s hypothesis that mitochondrial function in tumor cells is irreversibly damaged, it has been firmly demonstrated that mitochondria-mediated function is absolutely essential for tumor cells^7^. However, the exact functions of mitochondrial metabolism in cancer cells and the underlying molecular mechanisms remain poorly documented.

The peroxisome proliferator-activated receptor γ coactivator-1α (PPARGC1A, named hereafter PGC1α) is a member of the PGC1 family of coactivators^8^. PGC1α acts as one of the central transcriptional regulators of gene networks involved in mitochondrial biogenesis and respiration^9^. The mitochondrial electron transport chain (*ETC*), especially complex I and III, is the main producer of reactive oxygen species (ROS);^10^ therefore, the increased mitochondrial content and enhanced mitochondrial respiratory activity stimulated by PGC1α could in principle lead to an increase in the generation of ROS. Concurrently, PGC1α is known to be able to potently upregulate ROS-detoxifying enzymes including glutathione peroxidase and manganese SOD to protect cells against oxidative stress^11^.

To date, it is incompletely understood what effect this seemingly paradoxical regulation of PGC1α on ROS metabolism would have on the redox status of CCA. It is also unknown how PGC1α regulates glucose metabolism and ROS metabolism in CCA and what are the growth and metastasis consequences this might cause to CCA.

## Results

### PGC1α expression was downregulated in CCA tissues and cell lines

**Fig. 1:**
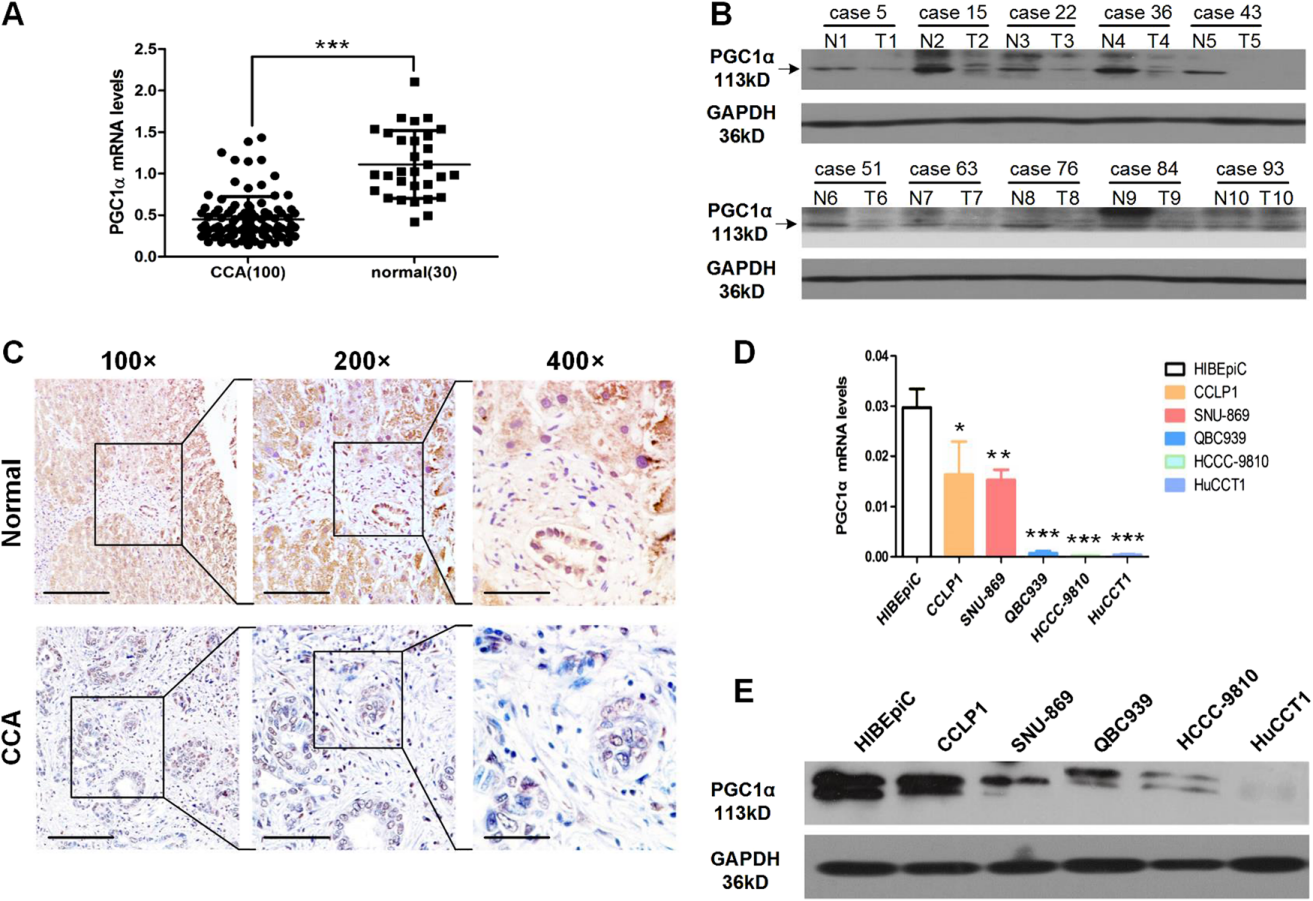
PGC1α is downregulated in human CCA tissues and cell lines. The samples were from our patients. **a** PGC1α mRNA levels in CCA tissues or normal bile duct tissues. **b** PGC1α protein levels in CCA tissues or adjacent nontransformed tissues. T: tumors; N: adjacent nontransformed tissues. **c** Representative images of PGC1α IHC staining in normal bile duct tissues and CCA tissues. Scale bars, × 40 : 1 mm; × 100 : 400 μm; × 400 : 100 μm. **d** Relative PGC1α mRNA levels in HIBEpiC and five CCA cell lines by qRT-PCR. **e** PGC1α protein levels in HIBEpiC and 5 CCA cell lines by western blot. All bar graphs are plotted as mean ± SD of three independent experiments performed in triplicate. *P < 0.05, **P < 0.01, ***P < 0.001

The qRT-PCR results indicated that the PGC1α mRNA levels were highly suppressed in CCA tissues compared with normal bile duct samples (Fig. 1a). PGC1α protein levels were verified to be markedly lower in CCA samples than in nonmalignant counterparts by western blot (Fig. 1b). Accordingly, the immunohistochemistry (IHC) results showed that PGC1α exhibited weak or negative staining in CCA samples with a rate of 69% (69/100), whereas in normal bile duct tissues the rate of weak or negative PGC1α staining was only 16.7% (5/30) (Fig. 1c). Regarding different cell lines, the PGC1α mRNA and protein levels detected in HIBEpiC cells, the normal human intrahepatic biliary epithelial cell line, were higher than those in five CCA cell lines (Fig. 1d,e). Subsequently, three CCA cell lines with very high levels (CCLP1), moderate levels (HCCC-9810) or very low levels (HuCCT1) of PGC1α mRNA and protein expression were chosen for further experiments. Together, all these results demonstrate that PGC1α levels are reduced in CCA tissues and cell lines.

### PGC1α has no impact on CCA proliferation but promotes CCA metastasis

**Fig. 2:**
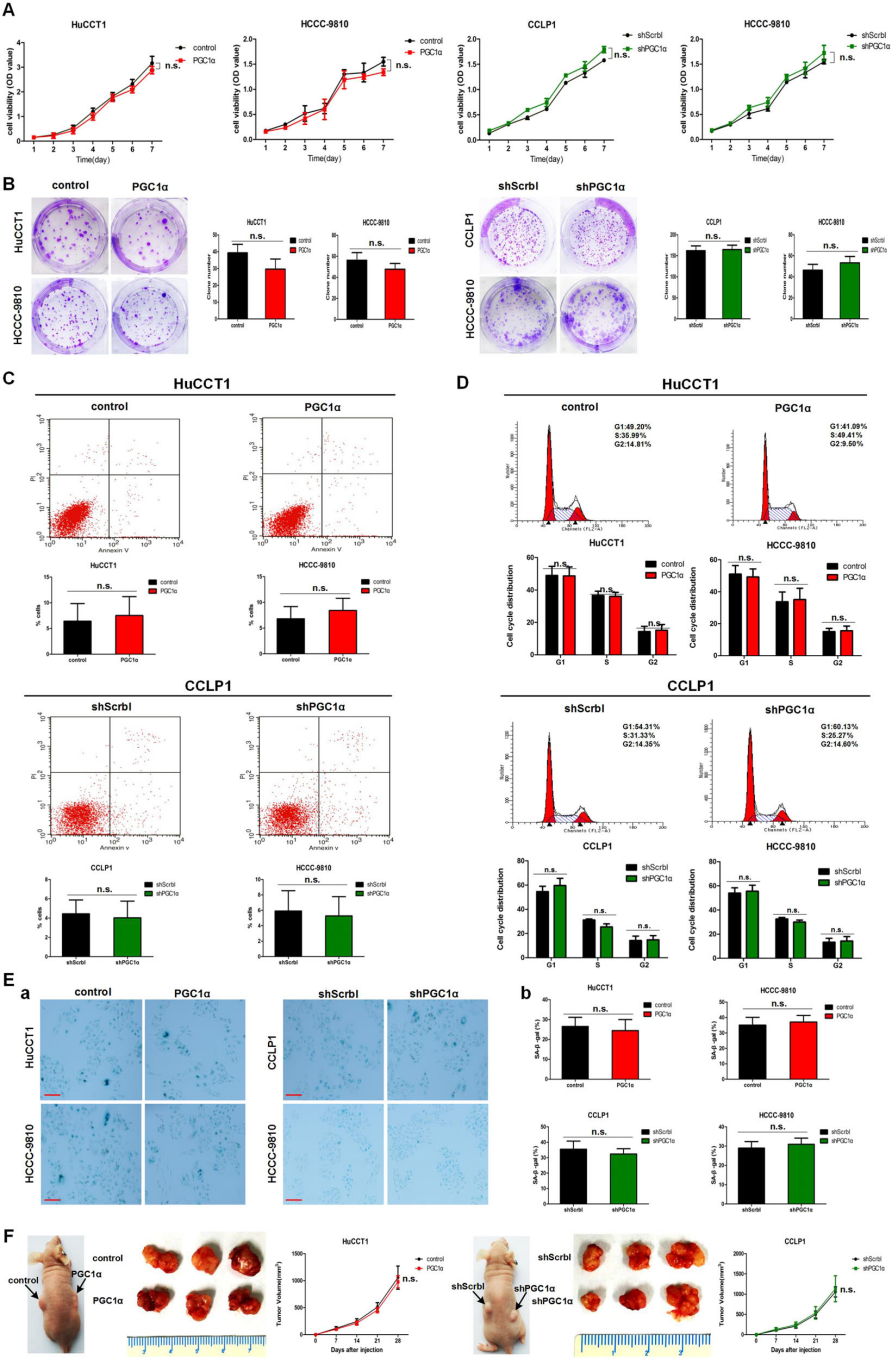
PGC1α has no effect on CCA cell proliferation and tumorigenesis. **a** Growth curves for the indicated CCA cells evaluated by the CCK-8 assays. **b** Representative images of the colony formation assays and analysis results. **c** Cell apoptosis determined by Annexin V/PI staining. **d** Representative images of the cell cycle analysis and analysis results of indicated CCA cells. **e** a Representative images of SA-β-gal staining in the indicated cells. Scale bars: 100 μm. b The quantification of positively SA-β-gal staining cells. **f** Tumor growth of HuCCT1 (*n* = 10) and CCLP1 (*n* = 10) xenografts and representative xenograft tumor images. The tumor growth curves are plotted as mean ± SD (*n* = 10, each group). All line graphs and bar graphs are presented as mean ± SD of three independent experiments performed in triplicate except otherwise stated. n.s. represents no statistical significance

To assess the role of PGC1α in CCA cells proliferation, we transfected PGC1α-expressing lentiviral vectors or lentiviral-based shRNA against PGC1α (shPGC1α) into CCA cells. Both the overexpression and knockdown of PGC1α were confirmed by qRT-PCR and western blot (Supplementary Figure 1). Cell growth assays showed that PGC1α overexpression and knockdown did not affect the viability of the indicated CCA cells over the course of 7 days (Fig. 2a). Accordingly, PGC1α did not affect colony number, cell apoptosis, cell cycle progression or cellular senescence (Fig. 2b–e) in CCA cells. Consistent with our in vitro data, PGC1α-transduced HuCCT1 tumors showed similar tumor growth kinetics and weight compared with HuCCT1-control tumors, and tumor size did not differ significantly between PGC1α-depleted cells and shScrbl cells (Fig. 2f).

**Fig. 3:**
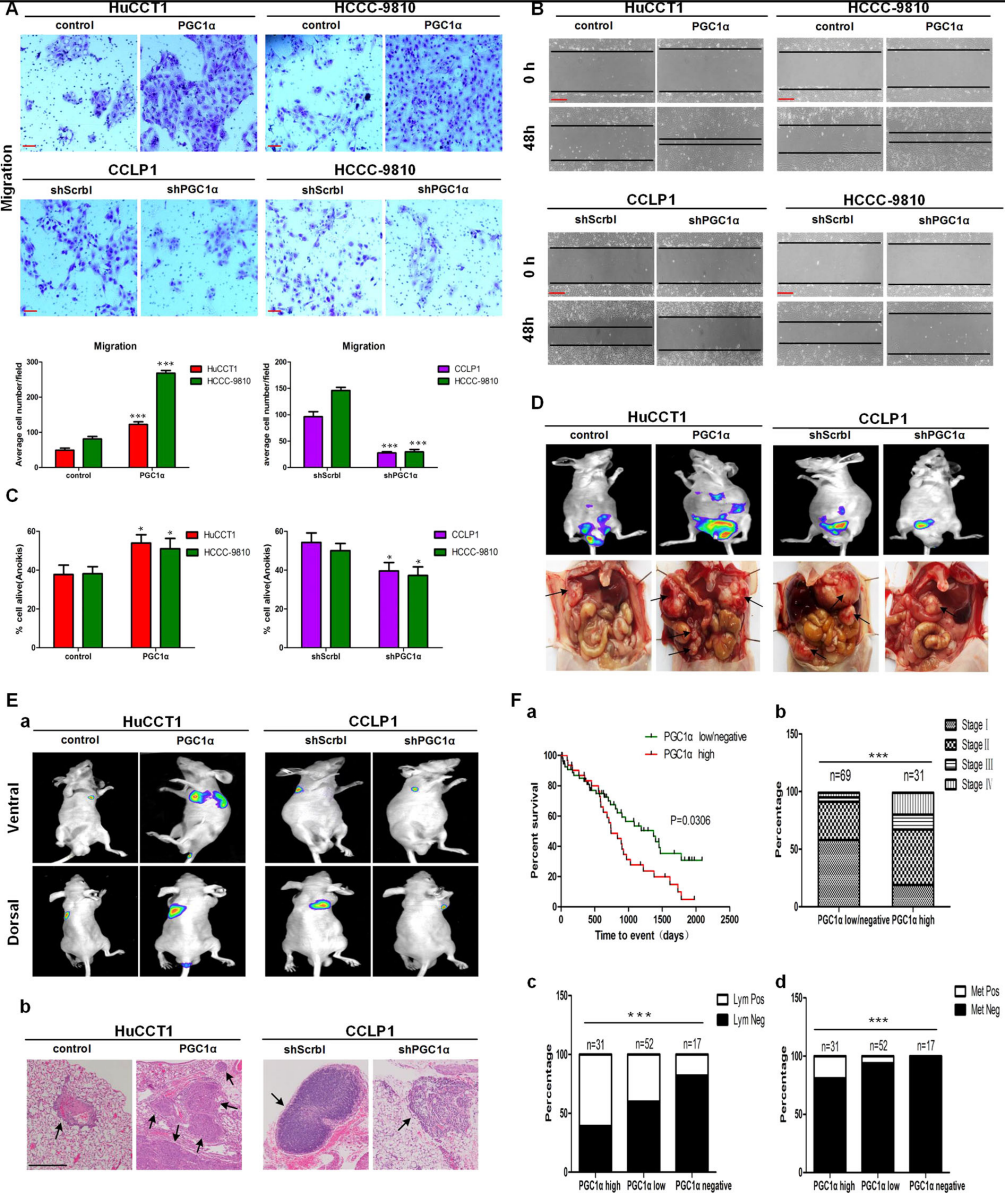
PGC1α facilitates metastasis of CCA. **a** Transwell migration assays and **b** wound-healing assays for the indicated cell lines. **a** Scale bars: 100 μm; **b** Scale bars: 500 μm. **c** Percent alive cells in anoikis assay. **d** Representative images of the tumor nodules in peritoneal cavity metastasis experiments. **e** a Representative bioluminescence images of lung metastases and b representative H&E images of lung metastases. Scale bars: 1 mm. **f** **a** Kaplan–Meier curve and b Percentage of patients with AJCC stage I–IV disease for CCA patients segregated by low/negative or high expression of PGC1α. c Percentage of patients with lymph node metastases and **d** Percentage of patients with distant metastases for CCA patients segregated by negative, low, or high expression of PGC1α. All bar graphs are presented as mean ± SD of three independent experiments performed in triplicate. *P < 0.05, ***P < 0.001

PGC1α has been reported to be involved in tumor metastasis without having effect on tumor growth^12^. Therefore, we next examined the effect of PGC1α on metastasis of CCA cells. Surprisingly, overexpression of PGC1α significantly increased the migratory and invasive capabilities of HuCCT1 and HCCC-9810 cells, and suppression of PGC1α diminished the migratory and invasive properties of CCLP1 and HCCC-9810 cells, as assessed by migration assays and wound-healing assays and invasion assays (Fig. 3a, b and Supplementary Figure 2A). In addition, as for CCLP1, HCCC-9810 and HuCCT1 cell lines, cells with higher endogenous levels of PGC1α displayed higher invasive capability (Supplementary Figure 2B). Furthermore, Higher anchorage-independent survival rates in cells overexpressing PGC1α was observed compared to control cells, and the percent of live cells was lower in shPGC1α cells than in shScrbl cells, assessed by anoikis assays (Fig. 3c).

Consistent with the *in vitro* data, the results of the peritoneal cavity metastasis models revealed that HuCCT1-PGC1α cells formed more metastatic nodules than HuCCT1-control cells, whereas the number of metastatic nodules was fewer in mice harboring CCLP1-shPGC1α cells than that in the control group (Fig. 3d). Correspondingly, the results of the lung metastasis models also showed that the volume of lung metastases were significantly increased in mice bearing HuCCT1-PGC1α cells than that in the control group, whereas mice implanted with CCLP1-shPGC1α cells had smaller lung metastases compared with the control group (Fig. 3e and Supplementary Figure 2C). Collectively, these results demonstrate that PGC1α affects neither CCA cells proliferation *in vitro* nor the primary tumor growth kinetics *in vivo* but does significantly promote CCA cells migration and invasiveness, both *in vitro* and *in vivo*.

Given the counterintuitive results, we next assessed the effect of PGC1α expression on clinical cases of CCA. The CCA patients were segregated into two or three groups based on the IHC results: a PGC1α-high group and a PGC1α-low or (and) -negative group. Surprisingly, there was a significant correlation between high PGC1α expression and a worse overall survival rate. Accordingly, patients whose tumors exhibited relatively high expression of PGC1α were significantly more likely to have advanced-stage tumors and metastatic disease (Fig. 3f). These results illustrate that relatively high PGC1α expression positively correlates with poor prognosis in CCA patients.

### PGC1α enhances pyruvate oxidation metabolism in CCA cells

**Fig. 4:**
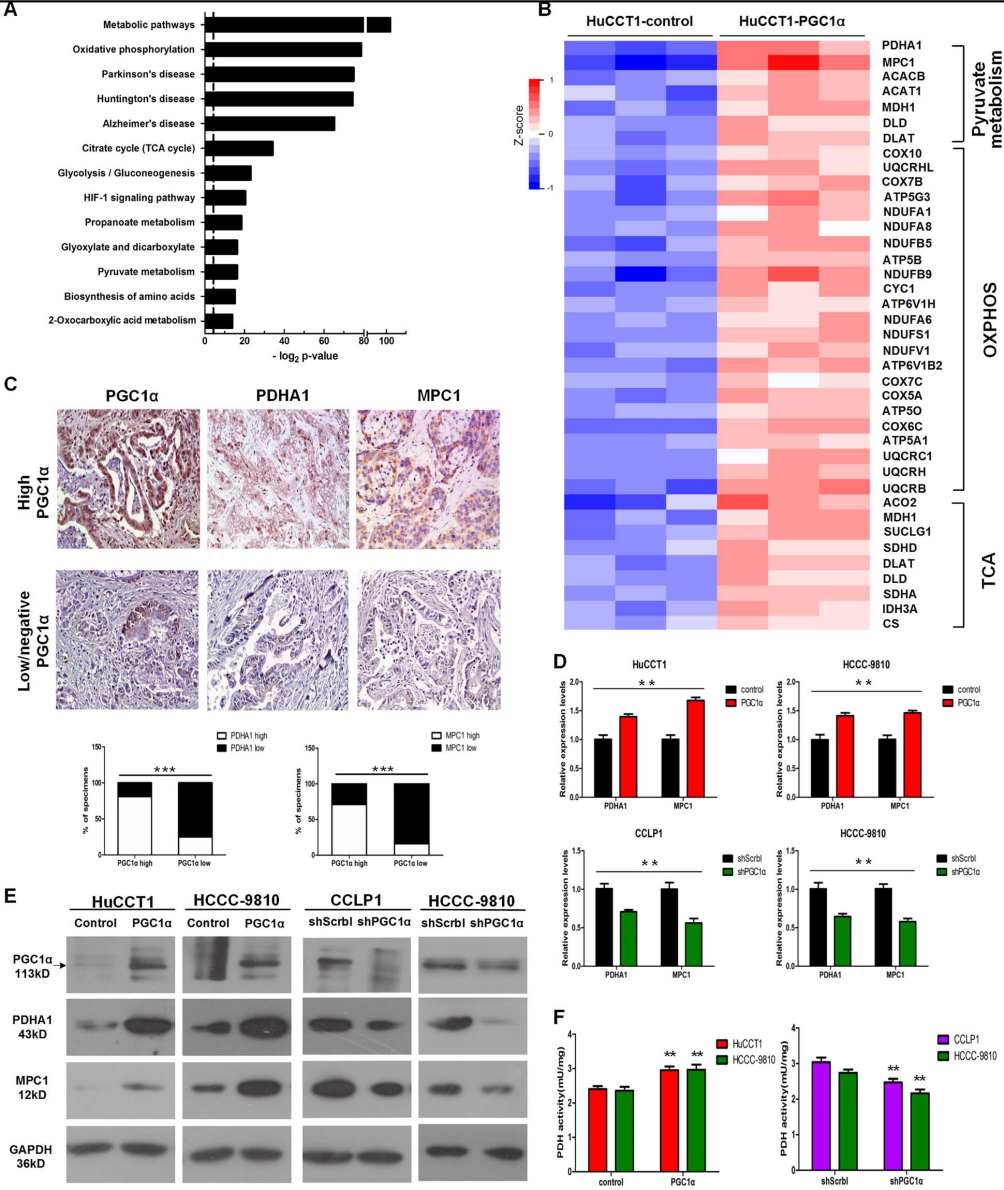
PGC1α regulates pyruvate metabolism. **a** KEGG (Kyoto Encyclopaedia of Genes and Genomes) analysis of the transcriptional program induced by PGC1α. The dotted line indicates *P* = 0.05. **b** Heatmap of differentially regulated genes in the pyruvate metabolism, OXPHOS, and TCA cycle gene set in HuCCT1-control and HuCCT1-PGC1α cells. **c** The expression of PGC1α is positively associated with the expression of PDHA1 and MPC1 in clinical CCA specimens. Representative IHC staining and result analysis. Scale bars, × 100 : 400 μm. **d** mRNA expression and **e** protein expression of PDHA1 and MPC1 in the indicated cells with PGC1α overexpression or knockdown. **f** PDH activity assay with PGC1α overexpression or knockdown. All bar graphs are presented as mean ± SD of three independent experiments performed in triplicate. **P < 0.01. ***P < 0.001

We next explored the transcriptional program associated with the metastasis promoting activity of PGC1α. Gene expression profiling coupled with bioinformatic analyses revealed that the metabolic pathways were the most differentially modulated canonical pathways in HuCCT1-PGC1α cells compared with HuCCT1-control cells. Notably, among the top 20 significantly altered pathways, most were related to mitochondrial metabolism, including pyruvate metabolism, the TCA cycle and oxidative phosphorylation (OXPHOS) (Fig. 4a). The heatmap unveiled a significant increase in transcription levels related to mitochondrial metabolism in HuCCT1-PGC1α cells. Interestingly, two key genes related to pyruvate metabolism reached a significant increase (>1.5-fold), including pyruvate dehydrogenase E1 alpha 1 subunit (PDHA1) and mitochondrial pyruvate carrier 1 (MPC1) (Fig. 4b). PDHA1 encodes the E1 alpha 1 subunit of PDH complex, which contains the E1 active sites and has a critical role in the function of PDH complex. MPC1 is a gate-keeping mitochondrial protein that control the entry of pyruvate into the mitochondria. Therefore, we speculated that PGC1α could reverse the Warburg effect by upregulating the expression of PDHA1 and MPC1 and simultaneously promote gene expression of the TCA and OXPHOS, thus enhancing mitochondrial metabolism.

In support of our hypothesis, by analyzing gene expression in CCA samples from The Cancer Genome Atlas data sets, we found that the expression levels of PDHA1 and MPC1 were positively correlated with PGC1α in CCA samples (Supplementary Figure 3). Furthermore, in clinical samples, 80.65% (25 cases) and 70.97% (22 cases) of samples with high PGC1α expression (31 cases) exhibited high level of PDHA1 and MPC1 respectively, whereas 75.36% (52 cases) and 84.06% (58 cases) of samples with low/negative PGC1α expression (69 cases) showed low expression of PDHA1 and MPC1 respectively (Fig. 4c). The mRNA and protein levels of PDHA1 and MPC1 were elevated following PGC1α overexpression (Fig. 4d,e). Accordingly, PDH enzyme activity was increased in PGC1α-overexpressing cells compared with control cells (Fig. 4f).

### PDHA1 and MPC1 are direct transcriptional targets of PGC1α/NRF1

**Fig. 5:**
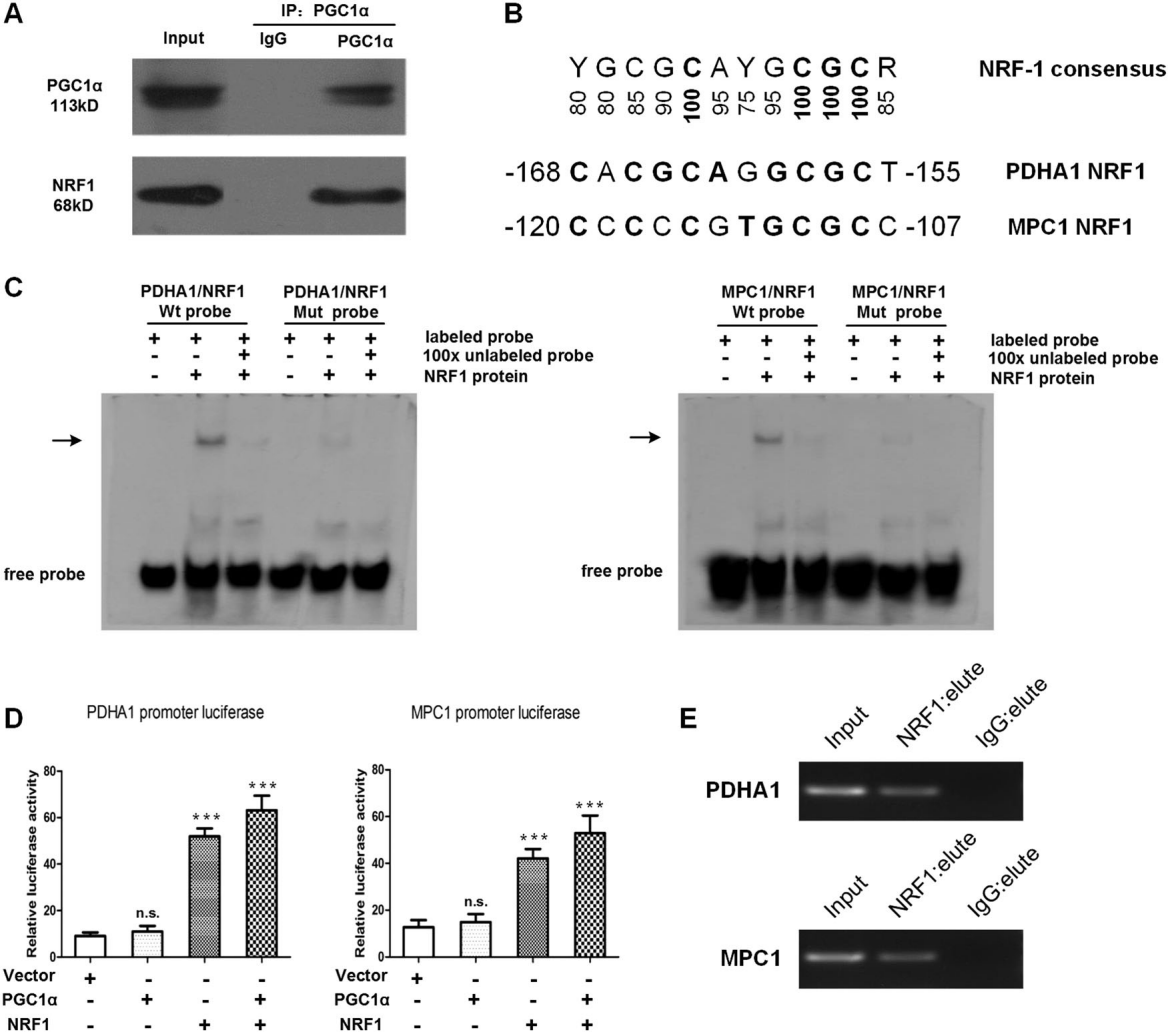
PGC1α/NRF1 complexes directly bind to and activate PDHA1 and MPC1 promoters. **a** Results of the co-IP assays shows that PGC1α forms a complex with NRF1 in HuCCT1- PGC1α cells. **b** NRF1 sites from PDHA1 and MPC1 are compared with the consensus. **c** EMSA were performed with nuclear extracts and radiolabeled probes encompassing the candidate NRF1-binding sequence on the PDHA1 or MPC1 promoter. **d** The effect of PGC1α and NRF1 on transcriptional activity of PDHA1 and MPC1 promoters, measured by luciferase reporter assays. Data are the mean ± SD of three independent experiments performed in triplicate. **e** ChIP assay showing the binding of NRF1 to PDHA1 or MPC1 promoter *in vivo*. The promoter regions of PDHA1 or MPC1 were amplified using the input and immunoprecipitated DNA as templates. ***P < 0.001

Next, we hypothesized that PGC1α transactivates PDHA1 and MPC1 gene transcription by binding to a transcription factor (TF). We analyzed the PDHA1 and MPC1 promoter sequences using the JASPAR, PROMO and SWISSREGULON algorithms and found that NRF1 is a common TF. More importantly, NRF1 has been linked to the transcriptional control of many genes involved in mitochondrial function and biogenesis^13^. Therefore, we speculated that the TF most likely to interact with PGC1α was NRF1. The interaction between PGC1α and NRF1 was confirmed by co-immunoprecipitation (co-IP) assays (Fig. 5a). We found putative NRF1-binding sites on the PDHA1 or MPC1 promoter by the JASPAR algorithm. As shown in Fig. 5b, the PDHA1 and MPC1 NRF1 sites are near-perfect matches to the NRF1 consensus. Electrophoresis mobility shift assays (EMSA) indicated that DNA–protein complexes were formed using crude nuclear extracts of HuCCT1-PGC1α cells and radiolabeled PDHA1 and MPC1 NRF1 oligomers (Fig. 5c). Next, the candidate binding sites of the promoters were cloned at 5′ of the SV40 promoter and fused to the luciferase reporter. To the end, PGC1α and NRF1 induced a significant increase in the activity of luciferase reporter carrying PDHA1 or MPC1 promoter fragment (Fig. 5d). Accordingly, chromatin immunoprecipitation (ChIP) assays showed that the promoter regions of PDHA1 and MPC1 were amplified from the DNA recovered from the immunoprecipitation complex using NRF1 antibody (Fig. 5e). Taken together, we first demonstrate that PGC1α/NRF1 complexes can directly bind to the PDHA1 and MPC1 promoters to promote gene transcription.

### PGC1α expression reverses the Warburg effect, mediating the metabolic switch from aerobic glycolysis to OXPHOS

**Fig. 6:**
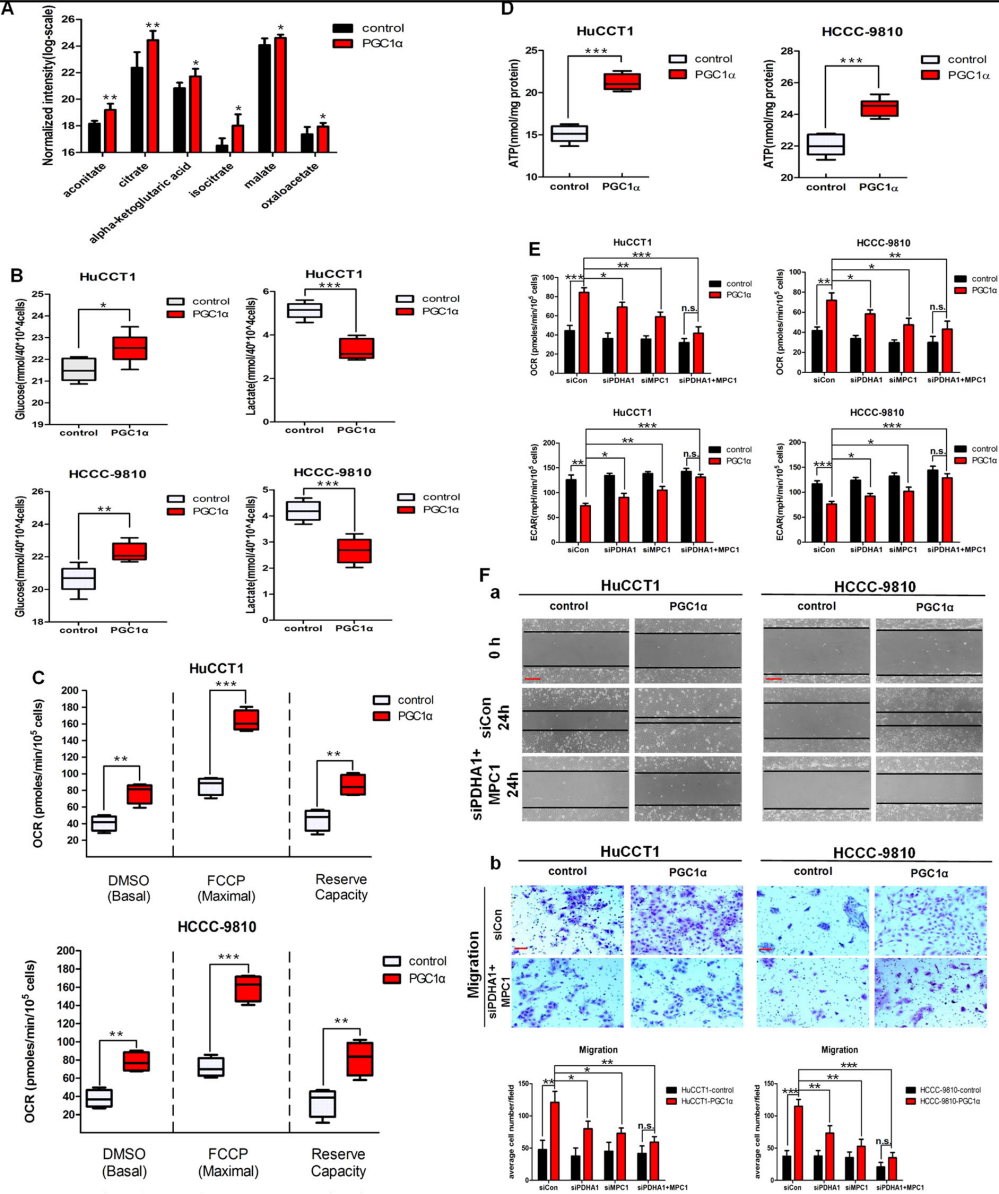
PGC1α facilitates TCA and OXPHOS. **a** LC–MS/MS measurements show the abundance of TCA intermediates in HuCCT1- PGC1α cells. **b** Glucose concentration and lactate concentration in control and PGC1α-overexpressing cells. **c** Basal and maximal OCR were measured in DMSO- or FCCP-treated control and PGC1α-overexpressing cells. **d** Intracellular ATP levels in control and PGC1α-overexpressing cells. **e** The effect of silencing of PDHA1 and MPC1 using siRNA on the OCR and ECAR in control and PGC1α-overexpressing cells. **f** The wound-healing and b migration assays after silencing PDHA1 and MPC1 in control and PGC1α-overexpressing cells. a Scale bars: 500 μm; b Scale bars: 100 μm. All bar graphs are presented as mean ± SD of three independent experiments performed in triplicate. All box plots are presented as mean ± SD of two independent experiments performed in quadruplicate. *P < 0.05, **P < 0.01, ***P < 0.001; n.s. no statistical significance

Liquid chromatography–mass spectrometry (LC–MS)/MS metabolomics validated that HuCCT1-PGC1α cells had increased levels of TCA intermediates (Fig. 6a). Subsequent biochemical assays also confirmed the decreased glucose uptake and decreased lactate production in PGC1α-transduced cells (Fig. 6b). We then measured the energy phenotype of the cells. PGC1α-overexpressing CCA cells exhibited substantially increased basal and maximal oxygen consumption rates (OCR) (Fig. 6c). We also observed a decrease in extracellular acidification rate (ECAR) in PGC1α overexpression CCA cells (Supplementary Figure 4A). Consistent with oxidative metabolism generating higher ATP levels than glycolytic metabolism, the intracellular ATP levels in PGC1α-overexpressing CCA cells were increased (Fig. 6d).

Conversely, depletion of PGC1α levels appears to be critical for enabling and promoting the Warburg effect in CCA cells, e.g., decreased basal OCR, increased ECAR, increased glucose uptake and lactate production, and reduced intracellular ATP levels (Supplementary Figure 4B-4F).

Furthermore, we explored whether the PGC1α overexpression-promoted CCA metastasis could be reversed by knockdown of PDHA1 and MPC1 in CCA cell lines. The silencing efficiency by siRNA was confirmed by qRT-PCR (Supplementary Figure 5A). We found that knockdown of PDHA1 and MPC1 reversed the avidity of PGC1α overexpression cells for OXPHOS (Fig. 6e) and blocked tumor cells migration and invasion mediated by PGC1α (Fig. 6f and Supplementary Figure 5B).

In aggregate, our results support that PGC1α reverses the Warburg effect by upregulating PDHA1 and MPC1 expression, thus enhancing mitochondrial metabolism and facilitating CCA cells migration and invasion.

### PGC1α promotes mitochondrial biogenesis and mitochondrial fusion to direct the metabolic shift

**Fig. 7:**
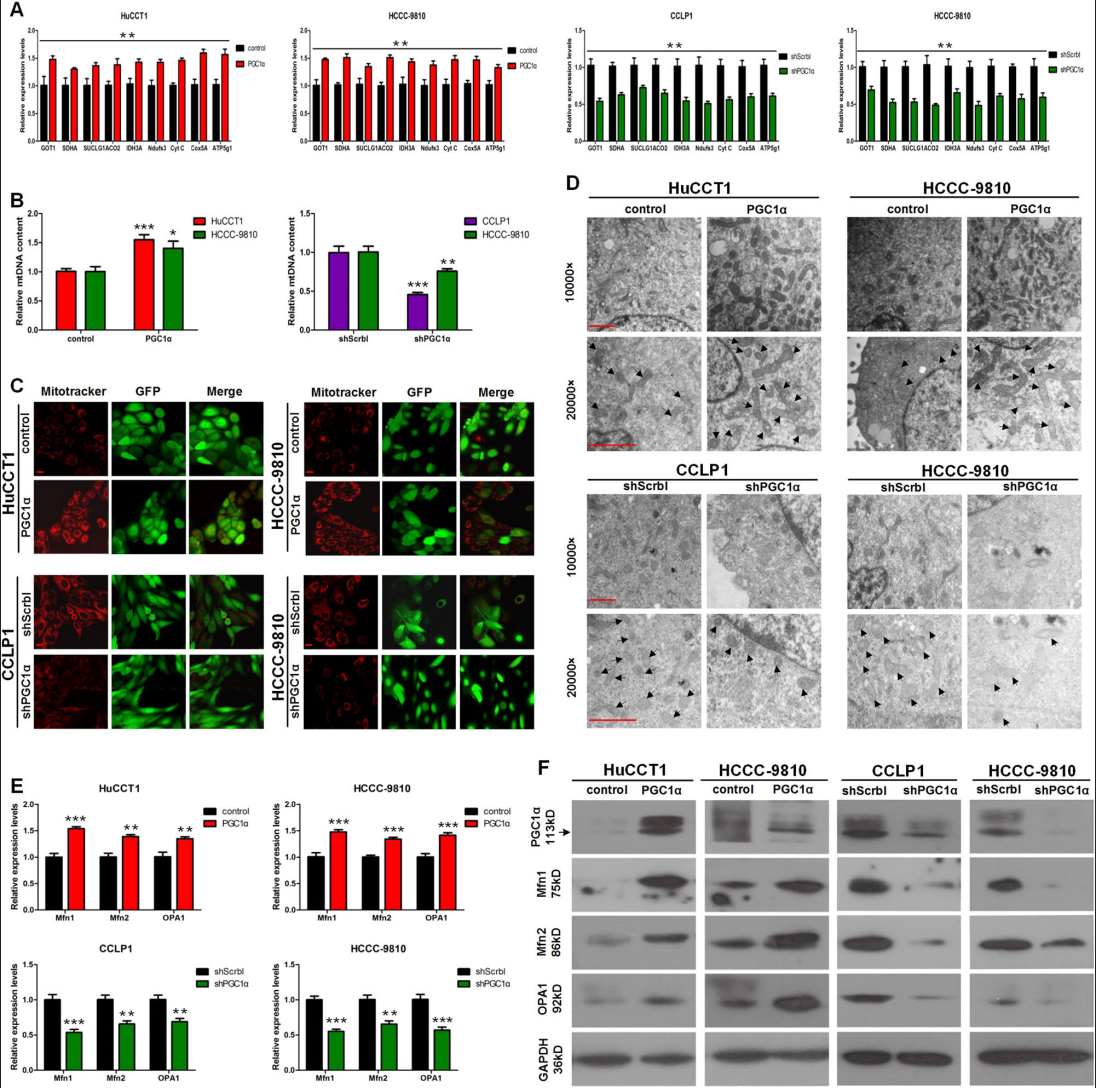
PGC1α promotes mitochondrial biogenesis and fusion. **a** mRNA levels of PGC1α mitochondrial target genes and **b** mtDNA measurements normalized to nuclear DNA in the indicated cells transduced with PGC1α or shPGC1α. **c** Mitochondrial mass of the indicated cells transduced with PGC1α or shPGC1α measured by MitoTracker Red CM-H_2_XRos staining. Scale bars: 20 μm. **d** Transmission electron microscopy of the indicated CCA cells with PGC1α overexpression or knockdown, black arrowheads identify mitochondria. Scale bars, both the upper and lower panel: 2 μm. **e** mRNA levels and **f** protein levels of Mfn1, Mfn2 and OPA1 in cells with PGC1α overexpression or knockdown. All bar graphs are presented as mean ± SD of three independent experiments performed in triplicate. *P < 0.05, **P < 0.01, ***P < 0.001

Corroborating microarray results and previous studies indicating that PGC1α has a central role in regulating the TCA cycle and OXPHOS, genes predominantly related to the mitochondrial TCA cycle and OXPHOS were significantly upregulated in PGC1α-overexpressing cells (Fig. 7a). Accordingly, the mitochondrial DNA copy number was elevated in PGC1α-overexpressing CCA cells and decreased in PGC1α knockdown CCA cells (Fig. 7b). Similarly, ectopic expression of PGC1α increased the fluorescence intensity of MitoTracker, an indicator of mitochondrial mass, whereas PGC1α knockdown cells exhibited weaker fluorescence intensity than shScrbl cells (Fig. 7c).

Mitochondria continually display a continuous cycle of fusion and fission, which has been linked to mitochondrial respiratory capacity^14^. Mitochondrial fusion is associated with high respiration capacity^15^. Considering that PGC1α powerfully promotes mitochondrial respiration, we hypothesized that PGC1α facilitated mitochondrial fusion to enhance OXPHOS. As expected, transmission electron microscopy showed that in addition to the increased number of mitochondria, mitochondrial volume was also significantly increased in PGC1α-overexpressing cells (Fig. 7d). Mitofusin-1(Mfn1), mitofusin-2 (Mfn2) isoforms, and optic atrophy 1 (OPA1) protein are three key proteins mediating mitochondrial fusion^16^. Interestingly, all three genes have previously been reported to be direct PGC1α/ERRα gene targets^17^. Indeed, the mRNA levels (Fig. 7e) and protein levels (Fig. 7f) of the three genes were significantly upregulated following the overexpression of PGC1α. Taken together, our findings converge to demonstrate that PGC1α drives mitochondrial biogenesis and fusion to boost OXPHOS capacity.

### PGC1α increases ROS production and promotes CCA metastasis

**Fig. 8:**
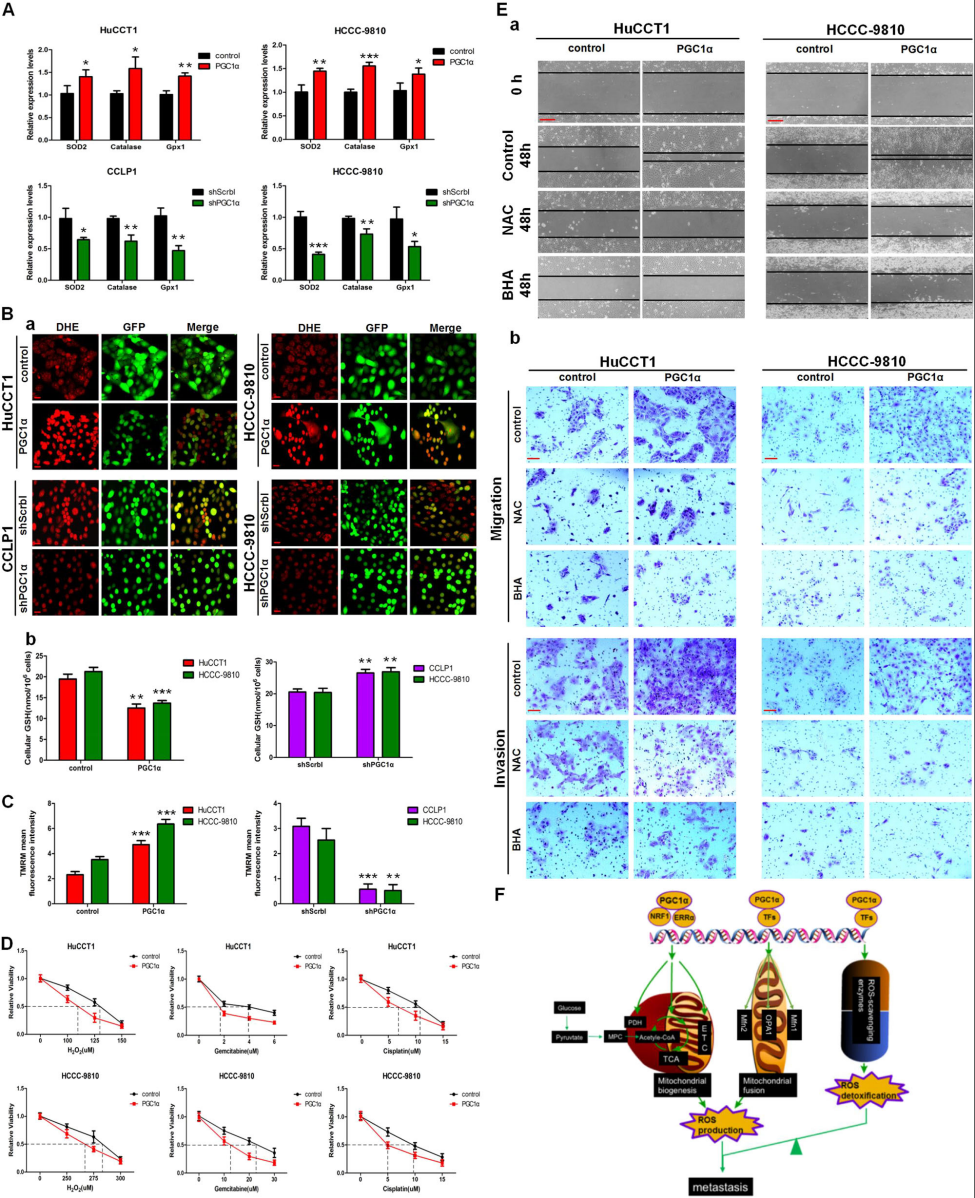
PGC1α facilitates CCA metastasis but simultaneously enhances sensitivity to cytotoxic therapies by increasing ROS levels. **a** mRNA levels of ROS detoxification enzyme genes in PGC1α or shPGC1α-transduced cells. **b** a ROS levels, detected by DHE fluorescent dye and b cellular GSH levels after overexpression or depletion of PGC1α. Scale bars: 20 μm. **c** Mitochondrial membrane potential, measured by TMRM staining in PGC1α overexpression or knockdown cells. **d** CCK-8 assay of control and PGC1α-transduced cells treated with the indicated dose of the indicated cytotoxic drugs for 48 h. **e** Representative images of a Wound-healing assays and b migration and invasion assays of the PGC1α overexpressing cells treated with NAC or BHA. a Scale bars: 500 μm; b Scale bars: 100 μm. **f** Schematic presentation of the pathway underlying PGC1α-facilitated CCA metastasis. All bar graphs are presented as mean ± SD of three independent experiments performed in triplicate. *P < 0.05, **P < 0.01, ***P < 0.001

In view of the dual regulation of PGC1α on ROS levels and the complex effect of ROS on cancer cells, we further investigated the role of ROS in the process of CCA metastasis induced by PGC1α. As reported previously, PGC1α expression did increase the expression of several antioxidant genes (Fig. 8a). However, dihydroethidium staining showed that in PGC1α-overexpressing cells, intracellular concentrations of ROS were increased, accompanied by a decrease in GSH levels (Fig. 8b), indicating that the antioxidant response was not sufficient to detoxify the increase in ROS. It has been reported that increased ROS generation could decrease mitochondrial membrane potential (Δψm) and sequentially trigger mitochondria-dependent apoptosis^18^. However, we did not observe a decrease of Δψm in PGC1α-overexpressing cells. Instead, we found a higher fluorescence intensity in PGC1α-overexpressing cells with the Δψm-sensitive-positive dye tetramethylrhodamine methyl ester, reflecting increased ETC activity and mitochondrial function (Fig. 8c and Supplementary Figure 6A). In addition, we tested whether increased ROS levels would sensitize CCA cells to cytotoxic therapies. The results showed that increased ROS rendered PGC1α-overexpressing cells more sensitive to H_2_O_2_ and chemotherapeutic drugs gemcitabine and cisplatin (Fig. 8d). Subsequently, we investigated whether the antioxidants *N*-acetyl-*L*-cysteine (NAC) and butylated hydroxyanisole (BHA) could antagonize the metastatic phenotype of PGC1α. NAC and BHA abolished the increase in ROS levels (Supplementary Figure 6B) and blunted to varying extents the promotion effect of PGC1α on migration and invasion in HuCCT1 and HCCC-9810 cells (Fig. 8e and Supplementary Figure 7). Taken together, our data indicate that PGC1α leads to an increase in ROS levels and thus promotes CCA metastasis.

## Discussion

Our data showed that the PGC1α expression was reduced in CCA compared with noncancerous tissue. However, at odds with our expectations, PGC1α overexpression indeed tended to inhibit CCA cell growth but that was not statistically significant, suggesting that PGC1α possibly is not involved in growth signaling pathways. Intriguingly, we reveal that PGC1α promotes CCA metastasis with solid evidence for the first time. With regard to why the expression of PGC1α acting as an oncogene is suppressed in CCA, we speculate that PGC1α underexpression just reflects damage of mitochondrial metabolism in CCA cells, for PGC1α is an indicator for mitochondrial metabolism. The effect of enhanced mitochondrial metabolism on tumor growth is not clear^12,19,20^. However, most metastatic cancer cells depend on mitochondrial metabolism to produce energy^21,22^. Our data also support that CCA cells with relatively more active mitochondrial metabolism are more likely to metastasize. Possibly it also depends on which kind of cancer and which organ site cancer cells will colonize to, for instance, liver metastatic breast cancer cells showed increased glycolysis activity by activating PDK1 and thus inhibiting PDH^23^.

One major disadvantage of our study is that we find it difficult to demonstrate PGC1α expression is higher in metastatic CCA than that in non-metastatic CCA. Our tissue samples are from patients with no detectable metastases preoperatively, whereas samples from those patients with distant metastasis are not available for there is no opportunity for surgery. Therefore, there might exist a selection bias related to sample collection and it is difficult to compare PGC1α expression difference between matastatic CCA and non-metastatic CCA owing to the small number and lack of representation of metastatic samples.

There is an important dichotomy with respect to the pro- or anti-tumorigenic effects of PGC1α in different cancer types^20,24^ and even within a cancer type^19,25^, so is the impact of PGC1α on metastasis^12,26,27^. Furthermore, the regulatory effect of PGC1α on the growth and metastasis of a cancer type also shows a discrepancy. For instance, PGC1α promotes prostate cancer growth but suppresses metastasis^28,29^. Our study and some other studies show that PGC1α has no impact on tumor growth but promotes metastasis^12^. The reasons for this heterogeneous response remain vague.

Some studies shows that successfully metastasizing cells undergo reversible metabolic changes from oxidative metabolism to glycolysis in an effort to reduce ROS production^30^. In contrast, it has been reported that mitochondrial respiration fuels tumor cell invasion^31,32^. Given the diverse outcomes of PGC1α on tumor phenotype, we postulate that it is likely dictated at least in part by the self-control balance of PGC1α between metabolic process and redox stress. Our data suggest that OXPHOS enhancement induced by PGC1α is accompanied by an increase in ROS levels in CCA cells. As PGC1α simultaneously upregulates antioxidant scavenging system of cancer cells, the ROS levels in CCA cells reach a higher equilibrium state where ROS is not completely scavenged but below a threshold. To the end, the increased ROS levels are not enough to deteriorate OXPHOS and induce mitochondria-mediated apoptosis but support metastatic dissemination of CCA cells (Fig. 8f). Similarly, in other studies, if PGC1α-positive cells exhibit remarkably increased ROS detoxification capacities, PGC1α predominantly enhances mitochondrial metabolism and thus facilitates tumor growth^19^. In contrast, if overly high ROS levels that cannot be appropriately counterbalanced by the cellular antioxidant defense system would induce oxidative stress and tumor growth suppression;^20^ in circulating cancer cells, excessive ROS levels would reduce mitochondrial function and thus limit distant metastasis^33^.

## Materials and methods

### Tissue samples collection

Between 2007 to 2015, 100 CCA and non-tumor adjacent specimens and 30 normal bile duct tissues were collected during surgery at the First Affiliated Hospital of Harbin Medical University. The histopathology diagnosis was based on the World Health Organization criteria. The clinical classification of tumors was defined according to the International Union against Cancer TNM classification system. Tumor differentiation was assigned according to the Edmondson grading system. Ethical approval was acquired from the Research Ethics Committee of the First Affiliated Hospital of Harbin Medical University with the informed consent of each patient. The detailed clinicopathologic characteristics of all CCA patients are listed in Supplementary Table 1.

### Microarray analysis

RNA was extracted from the HuCCT1 cell line using Trisol (Invitrogen) and submitted to Biolancet Technology, Ltd. (Beijing, China). Gene expression microarray was performed using the Affymetrix GeneChip Human Transcriptome Array 2.0 and analyzed using Affymetrix Transcriptome Analysis Console software. The heatmap was drawn using R software.

### C**o**-**immunoprecipitation** assay

Cells were washed twice with cold phosphate-buffered saline (PBS), scraped off, centrifuged and lysed in 300 *μ*l buffer containing a protease inhibitors cocktail (Sigma-Aldrich). After centrifugation and preclearing of the lysate by incubating with 20 μl of protein A/G beads (Santa Cruz Biotechnology, USA) for 1 h at 4 °C, PGC1α complexes were precipitated using anti-PGC1α antibody for 12 h and protein A/G beads for 2 h. Precipitates were washed four times with lysis buffer, denatured by boiling in loading buffer, and detected by western blotting.

### Luciferase activity assay

The potential NRF1 sites on the human PDHA1 or MPC1 promoter were predicted on JASPAR websites. The candidate NRF1-binding sites on the PDHA1 and MPC1 promoter were cloned in a SV40 promoter driving luciferase reporter plasmid. The plasmid together with a NRF1 expression vector and (or) PGC1α expression vector or a control plasmid was transfected into 293T cells. After 48 h, luciferase activity was measured on the dual luciferase reporter assay system (Promega). Expression levels of reporter genes were normalized with renilla luciferase activity.

### EMSA

The radiolabeled dsDNA probes encompassing the NRF1-binding site from the PDHA1 promoter or the NRF1-binding site from MPC1 promoter (wild-type probe) were used in the assays, and their mutants (mutant probe), were used to test specific binding of NRF1 to the two sites. The wild-type and mutated unlabeled PDHA1 and MPC1 dsDNA oligonucleotides were used for competition analysis. The oligonucleotides employed in the assays were as follows (mutated nucleotides were underlined): PDHA1/NRF1, 5′-CCACCTTCCCACGCAGGCGCTATCAAGCCC-3′ and 3′-GGTGGAAGG-GTGCGTCCGCGATAGTTCGGG-5′; PDHA1/NRF1 mut, 5′-CCACCTTCCC-ACGGAGTTACTATCAAGCCC-3′, 3′-GGTGGAAGGGTGCCTCAATGATAG-TTCGGG-5′; MPC1/NRF1, 5′-CGGGCCCCGCCCCCGTGCGCCACCGAGG-GC-3′ and 3′-GCCCGGGGCGGGGGCACGCGGTGGCTCCCG-5′; MPC1/NRF1 mut, 5′-CGGGCCCCGCCCCGGTTTACCACCGAGGGC-3′ and 3′- GCCCGGGGCGGGGCCAAATGGTGGCTCCCG-5′. A total of 10 ng nuclear extracts of HuCCT1- PGC1α cells were incubated with 1000 fmol of labeled probes or 100 pmol unlabeled probes, followed by adding gel loading buffer. The mixture was then electrophoresed on a 6.5% nondenaturing polyacrylamide gel.

### ChIP and PCR amplification

Formaldehyde fixation, cell lysis, and sonication were carried out as previously described^34^. In total, 1 μg rabbit anti-NRF1 (abcam; ab34682) or 1 μg nonspecific immunoglobulin G (Santa Cruz) were used to immunoprecipitate chromatin. Input and immunoprecipitated DNA were subjected to reversing cross-links and purifying. Then real-time PCR experiments were performed by KangChen Bio-tech, Shanghai, China. Primers flanking the NRF1 site on the PDHA1 promoter: sense, 5′-GCAATCCCACTAGGACACAACA-3′; anti-sense, 5′-ACACACAGAGACAAAACACCAAAG-3′. Primers flanking the NRF1 site on the MPC1 promoter: sense, 5′-CACAAACTGGCTTGTCTATCCT-3′; anti-sense, 5′-TTCTCAACATTAGTGACCCGC-3′; Primers for GAPDH (negative control): sense, 5′-TACTAGCGGTTTTACGGGCG-3′; anti-sense, 5′-TCGAACAGGAGGAGCAGAGAGCGA-3′. Primers for MEF2A (positive control), sense, 5′-ACCGAGAGGATAATTCAGTCCTG-3′; anti-sense, 5′-ACATCCGCGCACGGATC-3′. One third of the reaction was electrophoresed on a 5% polyacrylamide gel, followed by quantification with a PhosphoImager (Molecular Dynamics) and Image Quant software.

### LC–MS/MS metabolomics

The cells were washed twice with PBS and once with saline. After adding 1 ml of a solution of methanol/acetonitrile/water (2:2:1 volume), the cells were scraped off quickly and quenched in liquid nitrogen. Samples were then vortexed and processed by ultrasonication at 4 °C and incubated 1 h at −20 °C to pellet cell debris and proteins. Samples were subsequently centrifuged at 14,000 rpm for 15 min at 4 °C and supernatants were saved. Metabolite extractions were dried by SpeedVac with no heat. Samples were re-suspended by adding 100 µl of a solution of acetonitrile/water (1:1 volume) and then separated by hydrophilic interaction chromatography. Some metabolites were targeted in negative ion MRM mode. Metabolomics data analysis was done using Metaboanalyst software.

### Animal studies

Male BALB/c nude mice (5–6 weeks old) were obtained from Beijing Vital River Laboratory Animal Technology Co., Ltd., and housed in a specific pathogen-free environment. All animal experiments were conducted in accordance with standard protocols of the Institutional Animal Care and Use Committee of Harbin Medical University. For xenograft tumor growth, 3 × 10^6^ CCA cells suspended in 200 μl of PBS were injected into the flanks of mice (*n* = 10/group). Tumor size was measured with Vernier calipers weekly, and volume was calculated using the formula: volume = length × (width)^2^ × 0.52. The mice were monitored over 4 weeks for tumor formation. To evaluate long-distance lung metastasis, the cells were transfected with a lentivirus containing firefly luciferase, and then 3 × 10^6^ CCA cells suspended in 150 μl of PBS were injected into nude mice (*n* = 10/group) through the tail vein. D-Luciferin (Gold Biotechnology, Hopkinton, MA) was administered intraperitoneally at 100 mg/kg body weight, and bioluminescence was detected with a Berthold NIGHTOWL LB983 imaging machine. Mice were killed at 12 weeks, and the lung metastases were confirmed by H&E staining. To evaluate the peritoneal metastasis, 3 × 10^6^ CCA cells in 200 μl of PBS were injected into the intraperitoneal cavity of 6–8-week-old male BALB/c mice (*n* = 10/group). Mice were imaged at 4 weeks after injection and then killed, and the number of metastatic nodules was counted.

Details on other experimental procedures are described in the Supplementary Methods.

## Electronic supplementary material

Supplementary figure legends

Supplementary Figure 1

Supplementary Figure 2

Supplementary Figure 3

Supplementary Figure 4

Supplementary Figure 5

Supplementary Figure 6

Supplementary Figure 7

Supplementary table 1

Supplementary table 2

Supplementary Methods
