# Supplementary material for: PGC1α promotes cholangiocarcinoma metastasis by upregulating PDHA1 and MPC1 expression to reverse the Warburg effect
Source: Cell Death Dis. 2018 Apr 27;9(5):466. doi: 10.1038/s41419-018-0494-0 (PMC5919932; doi:10.1038/s41419-018-0494-0)
Supplement: Supplementary file 1 — Supplementary figure legends [file 41419_2018_494_MOESM1_ESM.docx]

**Supplementary Figure Legends**

**Supplementary Figure 1.** The verification of the transfection efficiency. (A) PGC1α mRNA levels and (B) PGC1α protein levels were examined after Lenti-PGC1α and Lenti-shPGC1α transfection in indicated CCA cell lines. Values on the graphs represent mean ± SD of three independent experiments performed in triplicate. **P < 0.01, ***P < 0.001.

**Supplementary Figure 2.** PGC1α promotes the invasion of CCA cells. (A) Matrigel invasive assays for the indicated cell lines. Scale bars: 100 μm. (B) Invasion assays for the indicated CCA cell lines. Scale bars: 100 μm. (C) Representative images of metastatic nodules in the lungs. Values on the graphs represent mean ± SD of three independent experiments performed in triplicate. *P < 0.05, ***P < 0.001.

**Supplementary Figure 3.** Coexpression analysis for PGC1α in CCA versus PDHA1 or MPC1. Plotted data are mRNA expression from TCGA datasets.

**Supplementary Figure 4.** PGC1α knockdown mediates the metabolic switch to glycolysis. (A) ECAR in control and PGC1α overexpression cells. (B) Basal OCR and (C) ECAR in shScrbl and PGC1α knockdown cells. (D) Glucose levels, (E) lactate levels and (F) intracellular ATP levels in shScrbl and PGC1α knockdown cells. All bar graphs are presented as mean ± SD of three independent experiments performed in triplicate. All box plots are presented as mean ± SD of two independent experiments performed in quadruplicate.The whiskers in the box plots represent the maximum and the minimum value. **P < 0.01, ***P < 0.001.

**Supplementary Figure 5.** Silencing PDHA1 or (and) MPC1 using siRNA inhibites PGC1α- overexpressing cells invasion. (A) PDHA1 and MPC1 levels determined by qRT-PCR in control and PGC1α overexpression cells after siRNA transfection. (B) The invasion assays after silencing PDHA1 and MPC1 in control and PGC1α overexpression cells. Scale bars: 100 μm. All bar graphs are presented as mean ± SD of three independent experiments performed in triplicate. *P < 0.05, **P < 0.01, ***P < 0.001; n.s. no statistical significance.

**Supplementary Figure 6.** Representative fluorescence images of the indicated cells. (A)Representative TMRM staining images in the indicated cell lines. Scale bars: 20 μm. (B) DHE fluorescence changes of PGC1α-overexpressing cells treated with NAC or BHA. Scale bars: 20 μm

**Supplementary Figure 7.** NAC and BHA could reverse the promotion effect of PGC1α on CCA cells migration and invasion. Analysis of migration and invasion assays data of the indicated cells treated with NAC or BHA. Values represent mean ± SD of three independent experiments performed in triplicate.**P < 0.01, ***P < 0.001; n.s. no statistical significance.

**Supplementary Table 1.** The relationship between PGC1α staining of IHC and clinicopathological features of CCA patients.

**Supplementary Table 2.** All the primers used in real-time PCR.
