# Supplementary figures and images for: PGC1α promotes cholangiocarcinoma metastasis by upregulating PDHA1 and MPC1 expression to reverse the Warburg effect
Source: Cell Death Dis. 2018 Apr 27;9(5):466. doi: 10.1038/s41419-018-0494-0 (PMC5919932; doi:10.1038/s41419-018-0494-0)

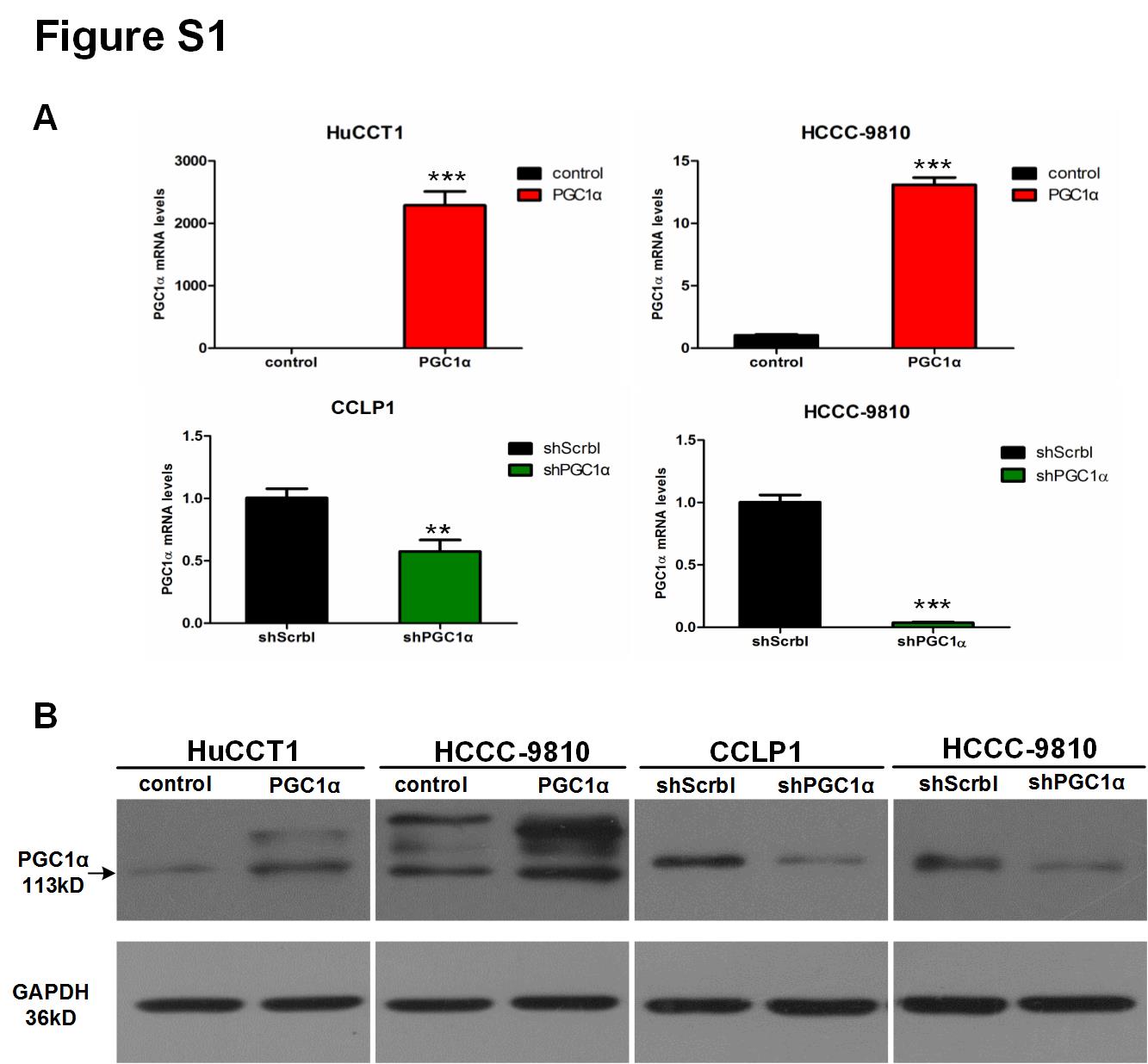

Supplement: Supplementary file 2 — Supplementary Figure 1 [file 41419_2018_494_MOESM2_ESM.jpg]

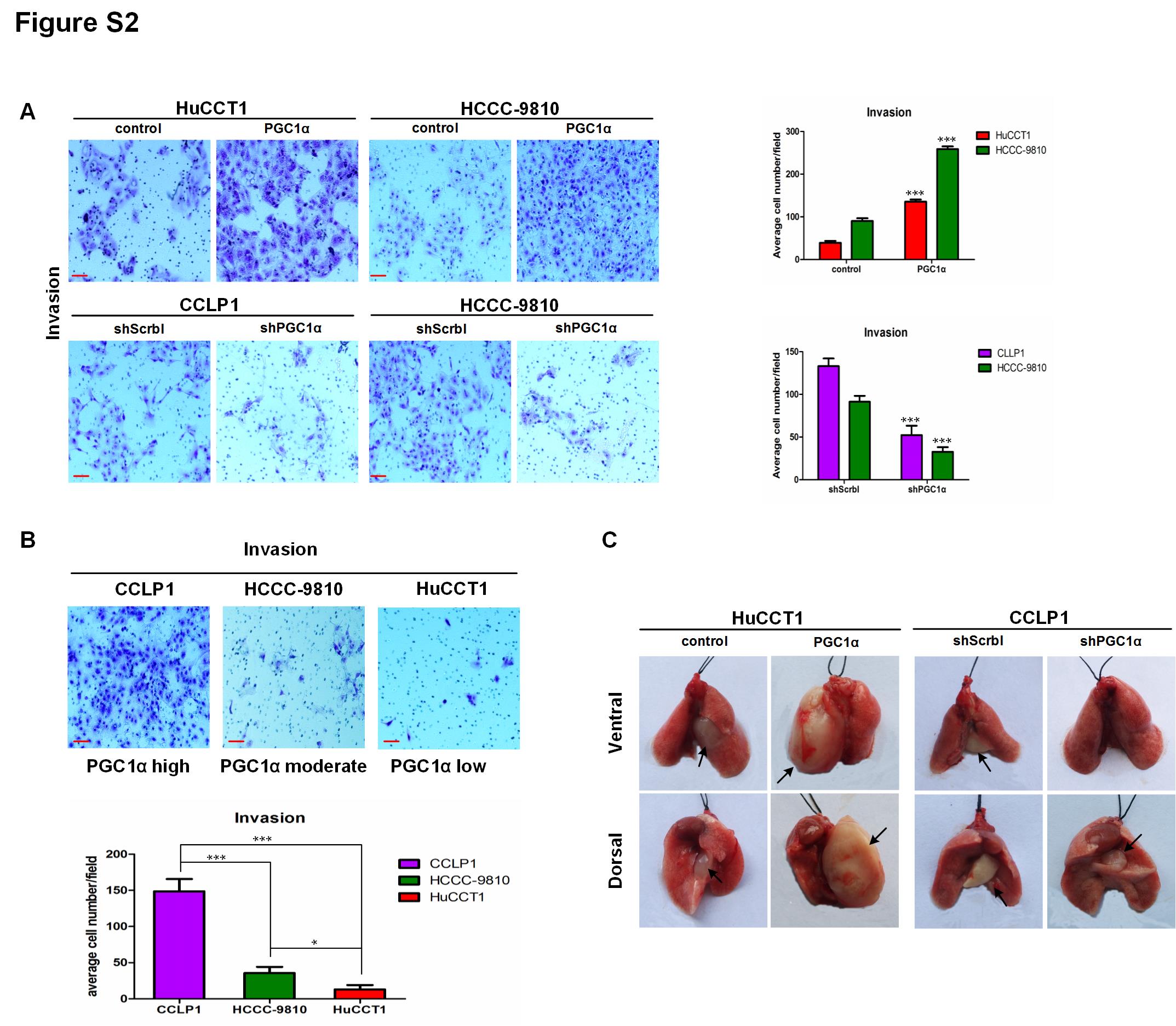

Supplement: Supplementary file 3 — Supplementary Figure 2 [file 41419_2018_494_MOESM3_ESM.jpg]

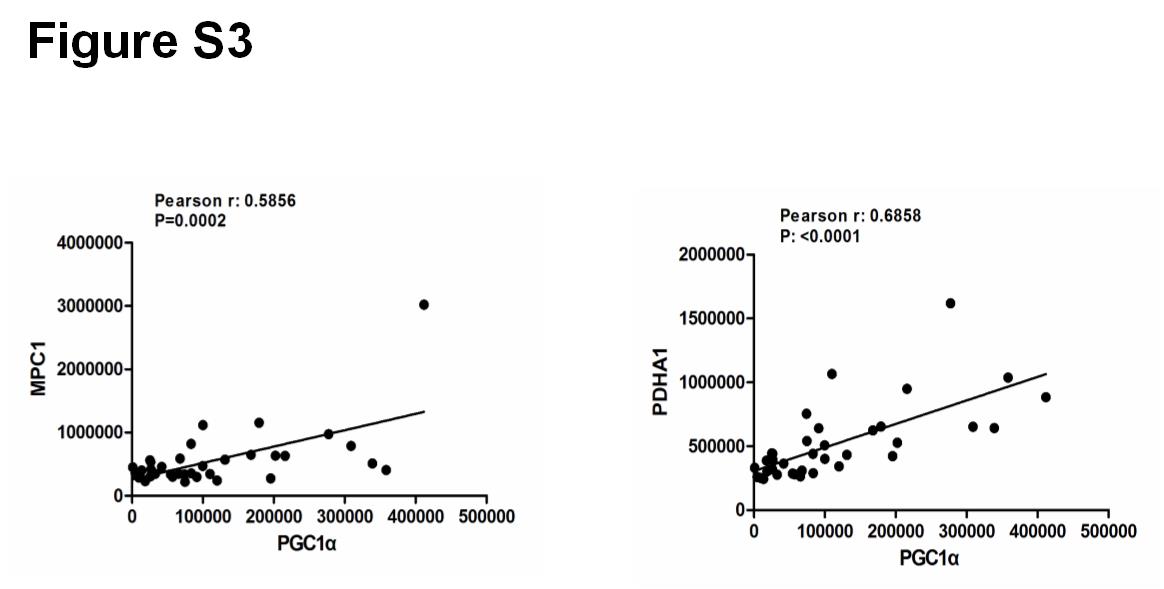

Supplement: Supplementary file 4 — Supplementary Figure 3 [file 41419_2018_494_MOESM4_ESM.jpg]

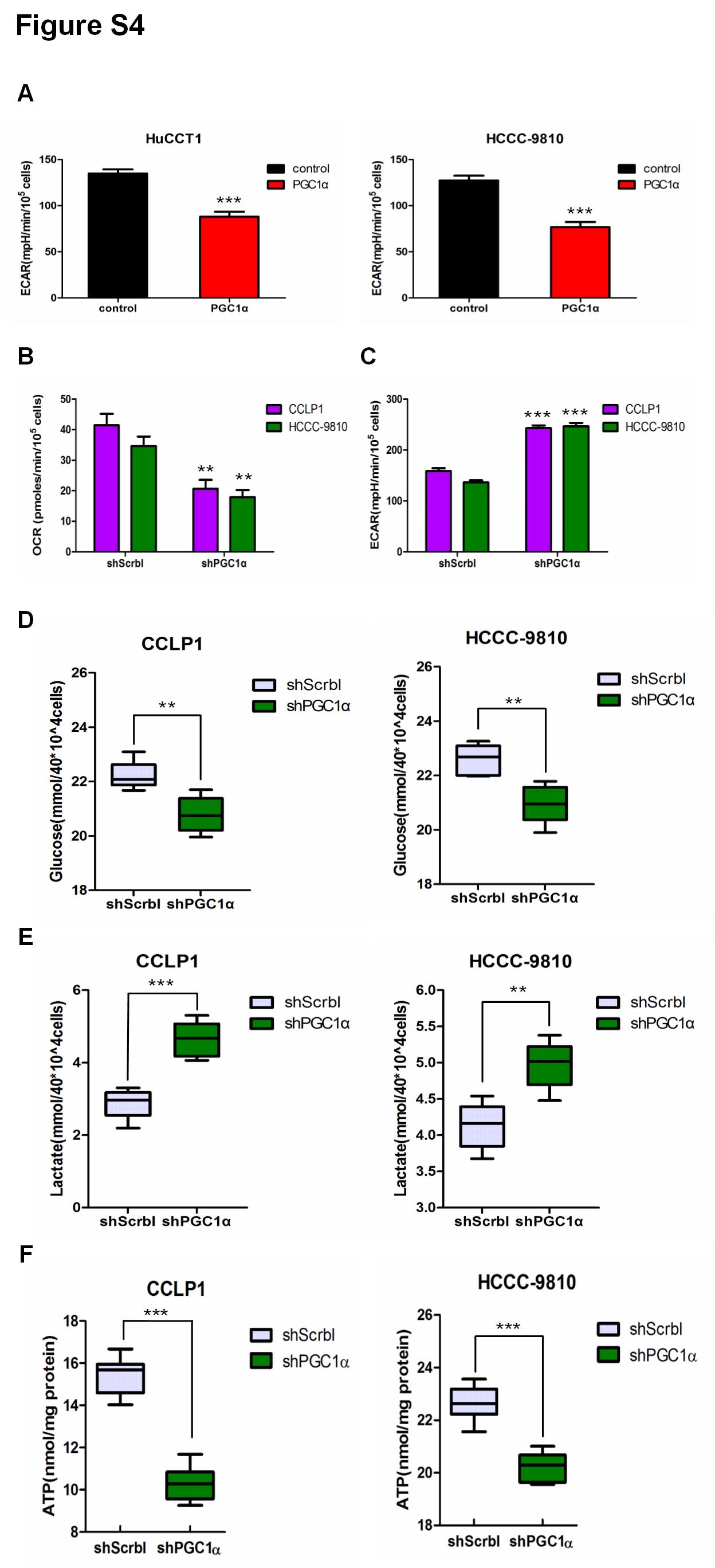

Supplement: Supplementary file 5 — Supplementary Figure 4 [file 41419_2018_494_MOESM5_ESM.jpg]

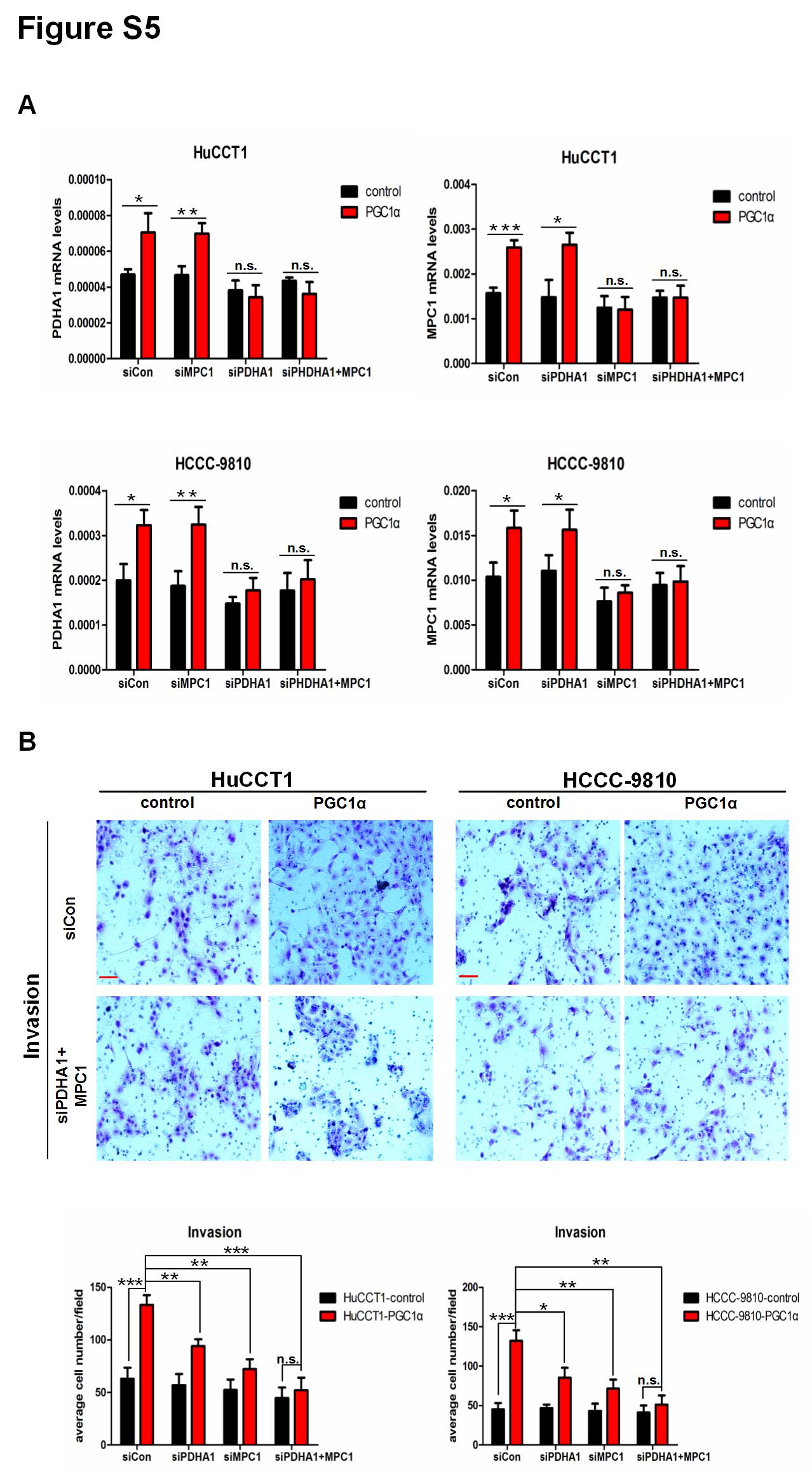

Supplement: Supplementary file 6 — Supplementary Figure 5 [file 41419_2018_494_MOESM6_ESM.jpg]

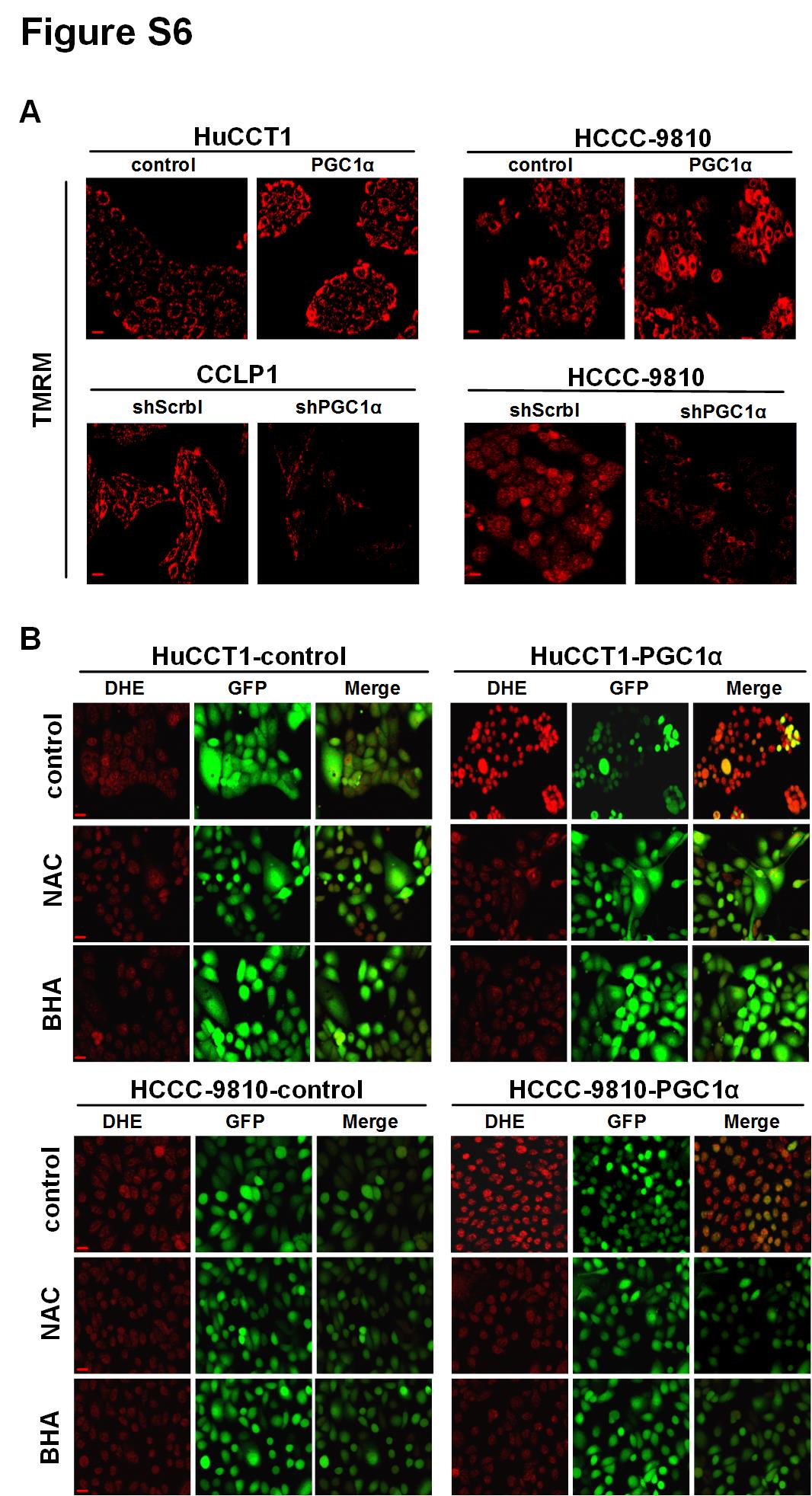

Supplement: Supplementary file 7 — Supplementary Figure 6 [file 41419_2018_494_MOESM7_ESM.jpg]

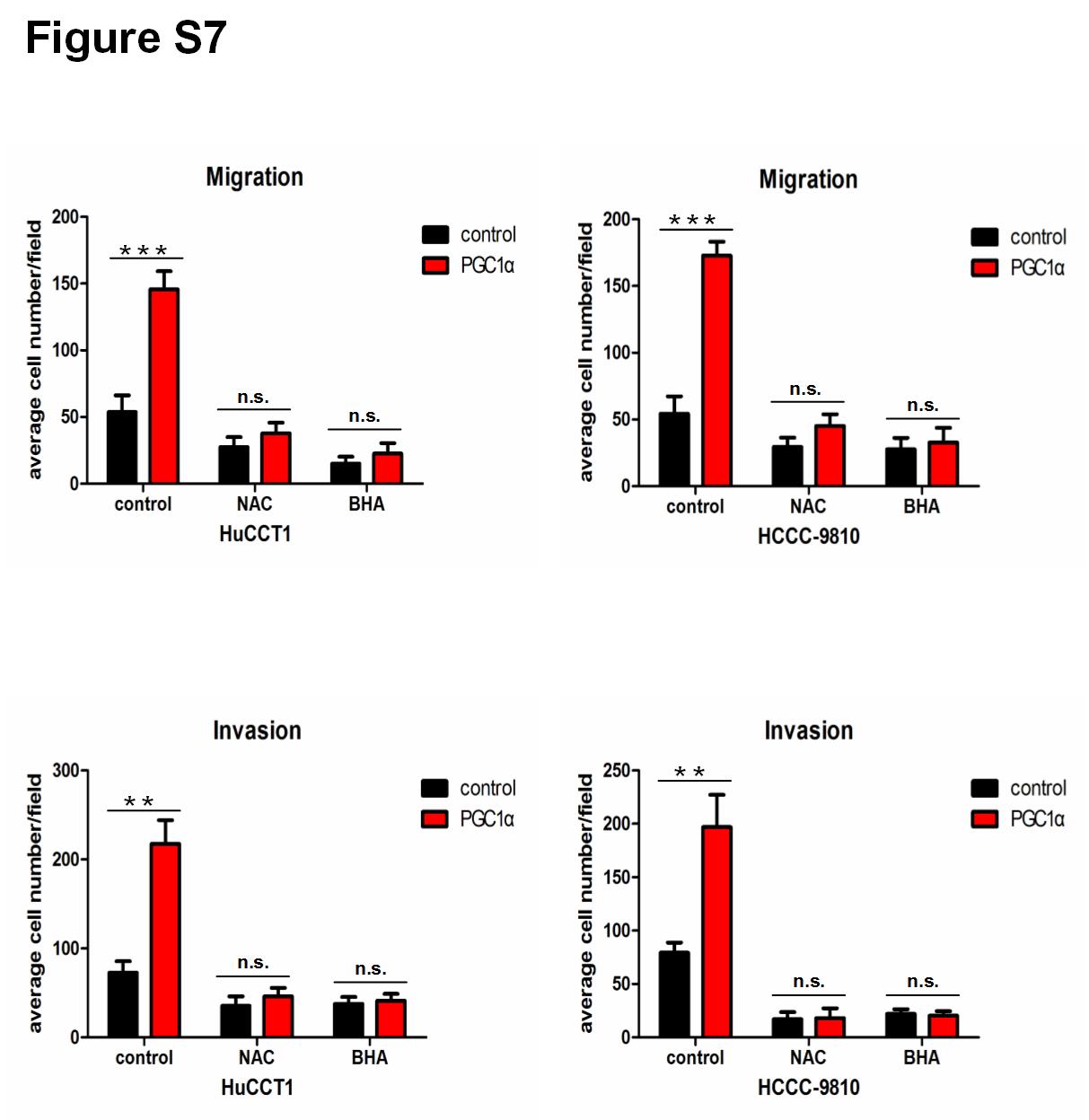

Supplement: Supplementary file 8 — Supplementary Figure 7 [file 41419_2018_494_MOESM8_ESM.jpg]
