# Supplementary material for: PGC1α promotes cholangiocarcinoma metastasis by upregulating PDHA1 and MPC1 expression to reverse the Warburg effect
Source: Cell Death Dis. 2018 Apr 27;9(5):466. doi: 10.1038/s41419-018-0494-0 (PMC5919932; doi:10.1038/s41419-018-0494-0)
Supplement: Supplementary file 9 — Supplementary table 1 [file 41419_2018_494_MOESM9_ESM.docx]

**Supplementary Table 1**

| Features | PGC1α staining | | P value |
| --- | --- | --- | --- |
|  | Positive(n=31)  n(%) | Low/negative(n=69)  n(%) |  |
| Age(years) |  |  |  |
| ≥60 | 18(58.06%) | 35(50.72%) | 0.3943 |
| <60 | 13(41.94%) | 34(49.28%) |  |
| Gender |  |  |  |
| Male | 20(64.52%) | 48(69.57%) | 0.5461 |
| Female | 11(35.48%) | 21(30.43%) |  |
| Histological differentiation | |  |  |
| Well | 8(25.81%) | 20(28.99%) | 0.6415 |
| Moderate | 13(41.94%) | 31(44.93%) |  |
| Poor | 10(32.26%) | 18(26.09%) |  |
| TNM stage |  |  |  |
| Ⅰ | 6(19.35%) | 40(57.97%) | <0.0001 |
| Ⅱ | 15(48.39%) | 23(33.33%) |  |
| Ⅲ | 4(12.90%) | 3(4.35%) |  |
| Ⅳ | 6(19.35%) | 3(4.35%) |  |
| CA19-9(U/ml) |  |  |  |
| ≤37 | 5(16.13%) | 16(23.19%) | 0.2842 |
| >37 | 26(83.87%) | 53(76.81%) |  |
| Lymph node metastasis |  |  |  |
| No | 10(32.26%) | 45(65.22%) | <0.0001 |
| Yes | 21(67.74%) | 24(34.78%) |  |
| Distant metastasis |  |  |  |
| No | 25(80.65%) | 66(95.65%) | 0.0014 |
| Yes | 6(19.35%) | 3(4.35%) |  |
