# Supplementary material for: PGC1α promotes cholangiocarcinoma metastasis by upregulating PDHA1 and MPC1 expression to reverse the Warburg effect
Source: Cell Death Dis. 2018 Apr 27;9(5):466. doi: 10.1038/s41419-018-0494-0 (PMC5919932; doi:10.1038/s41419-018-0494-0)
Supplement: Supplementary file 10 — Supplementary table 2 [file 41419_2018_494_MOESM10_ESM.docx]

| gene | Forward Primer  **Supplementary Table 2**  **Supplementary Table 2**  **Supplementary Table 2** | Reverse Primer |
| --- | --- | --- |
| PGC1α | AAAGGATGCGCTCTCGTTCA | CTTCAGCCTCTCGTGCTGAT |
| IDH3A | CTGCTCAGTGCCGTGATG | TCCTCTGTGAAGTCTGAGCATTT |
| Ndufs3 | GCTGACGCCCATTGAGTCTG | GGAACTCTTGGGCCAACTCC |
| Cyt C | GGAGGCAAGCATAAGACTGG | TCCATCAGGGTATCCTCTCC |
| Cox5A | GGGAATTGCGTAAAGGGATAA | TCCTGCTTTGTCCTTAACAACC |
| ATP5g1 | ATCATTGGCTATGCCAGGAA | ATGGCGAAGAGGATGAGGA |
| Complex II | CAAACCTACGCCAAAATCCA | GAAATGAATGAGCCTACAGA |
| CO1 | TGGAGCCTCCGTAGACCTAA | TGCGAAGCCTGGTAGGATAA |
| 16S rRNA (mtDNA) | GCCTTCCCCCGTAAATGATA | TTATGCGATTACCGGGCTCT |
| β2-microglobulin (nuclear DNA) | TGCTGTCTCCATGTTTGATGTATCT | TCTCTGCTCCCCACCTCTAAGT |
| MPC1 | GTGCGGAAAGCGGCGGACTA | GGCAGCAATGGGAAGACCCCA |
| PDHA1 | CTGGGCTGAGAGTGGATG | GTGGTAACGGTAAGTCTGC |
| SDHA | GGGAATGGTCTGGAACAC | AATCCGCACCTTGTAGTC |
| GOT1 | TCCGTCAGTCTTTGCCGAGGTTC | CGATATGCTCCCACTCCCAGGTTG |
| SUCLG1 | TACGAGTCAAGCACAAAC | CACAATGCCAATCCTTCC |
| ACO2 | AGGAAGGACATCAAGAAGG | GGCTGTGACAATCTCTGG |
| Mfn1 | GCGGAGACTTAGCATAATG | CGGATTCTTATATGTTGCTTC |
| Mfn2 | GTGCCAAGACTGTGAACC | TCAATACCAGGGCTGTCC |
| OPA1 | GATGGATCTGTGGATGCTGAAC | TTATCTGCTGAATCCTGCTTGG |
| SOD2 | TTGGCCAAGGGAGATGTTAC | AGTCACGTTTGATGGCTTCC |
| Gpx1 | CCCTCTGAGGCACCACGGT | TAAGCGCGGTGGCGTCGT |
| Catalase | TGTTGCTGGAGAATCGGGTTC | TCCCAGTTACCATCTTCTGTGTA |
| GAPDH | CATGAGAAGTATGACAACAGCCT | AGTCCTTCCACGATACCAAAGT |
