# Supplementary material for: PGC1α promotes cholangiocarcinoma metastasis by upregulating PDHA1 and MPC1 expression to reverse the Warburg effect
Source: Cell Death Dis. 2018 Apr 27;9(5):466. doi: 10.1038/s41419-018-0494-0 (PMC5919932; doi:10.1038/s41419-018-0494-0)
Supplement: Supplementary file 11 — Supplementary Methods [file 41419_2018_494_MOESM11_ESM.docx]

**Supplementary Methods:**

**Cell culture and virus infection**

HuCCT1 cell lines were kindly donated by Cancer Cell Repository, Tohoku University, Japan. HCCC-9810, SNU869, QBC-939 and CCLP1 were purchased from Shanghai Bioleaf Biotech Co., Ltd (Shanghai, China). The normal human intrahepatic biliary cell line (HIBEpiC) was purchased from ScienCell Research Laboratories (Carlsbad, CA). CCA cell lines were cultured in high glucose DMEM supplemented with 10% FBS and pen/strep. Lentiviral vector encoding the human PGC1α and empty vector were constructed in GV218(GeneChem, China). The lentiviruses scramble shRNA control (shScrbl)（5′ AAC AGT CGC GTT TGC GAC TGG 3′）and shRNA against PGC1α (shPGC1α)（5′CCA AGA CUC UAG ACA ACU ATT 3′）were constructed in GV-248(GeneChem, China). The transfection was performed with a multiplicity of infection (MOI) of 35 to 50 in the presence of polybrene (5 μg/ml). After lentiviral infection, single-cell clones were selected with 2.5 μg/ml puromycin (Sigma) for 2 to 4 weeks.

**Western blot**

Tissues and cells were lysed with RIPA buffer and proteins harvested. 40 μg of protein was separated using SDS-PAGE and transferred to PVDF membrane (Invitrogen). The following antibodies were used: PGC1α (Calbiochem), PDHA1(Abcam), MPC1(Cell Signaling Technology), OPA1(Abcam), Mfn1(Abcam), Mfn2(Abcam) and GAPDH (Sigma) antibodies. Proteins were visualized using ECL (SuperSignal Pierce Biotechnology) and quantified by scanning densitometry.

**Quantitative real-time polymerase chain reaction (qRT-PCR)**

Total RNA was extracted from cells and tissues using RNAeasy Mini Kit (Qiagen) and quantified with a Nanodrop ND-1000 spectrophotometer and reverse transcribed into cDNA using the High Capacity Reverse Transcription Kit (Applied Biosystems). Real-time PCR was performed using Power Sybr Green PCR Master Mix (Applied Biosystems) on an ABIPRISM 7500HT instrument (Applied Biosystems). The expression level of the indicated mRNAs was determined according to the ΔΔCt method using GAPDH as internal control. For mtDNA quantification, total DNA was extracted from cells using an E.Z.N.A™. Tissue DNA Kit (Omega, Atlanta, GA) following the manufacturer’s instructions. Primers were designed to amplify the cytochome oxydase 1 (CO1) , complex Ⅱ (succinate-ubiquinone oxidoreductase) and 16sRNA genes from the mtDNA genome and were normalized to the nuclear geneβ_2_-microglobulin. A complete list of primer sets can be found in Supplementary Table 2.

**Immunohistochemistry (IHC) analysis**

IHC staining was performed as we previously reported. ^1^  In brief, tissue sections were deparaffinized and rehydrated and then blocked with 10% normal goat serum. An anti-PGC1α antibody (Calbiochem) (1:500 dilution) or an anti-PDHA1 antibody (Abcam) (1:250 dilution) or an anti-MPC1 antibody (Abcam) (1:250 dilution) was used for incubation overnight at 4°C. The slides were then incubated at room temperature with secondary antibody (Vector lab, Burlingame, CA) for 1 h and the Vectastain Elite ABC reagent (Vector lab) for 30 min sequentially. Tissue sections were then stained with diaminobenzidine (DAB kit; Vector lab) and counterstained with hematoxylin (Sigma). The density of IHC staining was counted by Image-Pro Plus v6.2 software and the median value of density values of all the slides with positive staining was the cutoff to define high or low subgroups.

**Cell growth and colony formation assays**

For cell growth assays, 500-1000 cells were seeded onto 96-well plate. The optical density (OD) value was determined by adding CCK-8 solution and incubating for two hours at 37°C at indicated time points. For colony formation assays, 500-1000 cells were seeded into 6-well plates and cultured for 14 days. Then the colonies were fixed with 4% paraformaldehyde (PFA) and stained with 0.5% crystal violet to visualize colonies.

**Cell apoptosis analysis**

Cells were trypsinized with 0.25% trypsin containing no ethylenediaminetetraacetic acid (EDTA) and washed twice with PBS. 1 × 10^5^ cells were processed for annexin V/propidium iodide (PI) apoptosis detection (BD Biosciences) following the manufacturer’s instructions and then analyzed by flow cytometry (Beckman Coulter FC 500).

**Cell cycle analysis**

Cells were trypsinized, washed twice and then fixed in 95% ethanol at 4°C for 24 hs. After centrifuged and washed once, 1×10^6^ cells were stained following the instructions of Cycle TESTTM PLUS DNA Reagent Kit (BD Biosciences) and then measured by flow cytometry.

**Senescence-associated β-galactosidase (SA-β-Gel) staining**

SA-***β***-gal activity assays were performed with an SA-***β***-gal staining kit (Cell Signaling Technology) following the manufacturer’s instruction.

**In vitro wound-healing, migration and invasion assays**

For the wound-healing assay, 5-8×10^5^ CCA cells were cultured overnight and allowed to grow to confluence. Cells were gently scratched with a sterile 1000 μL pipette tip, washed three times with PBS and then incubated with the medium containing 0.5%FBS. Images were captured at 0 h, 12 hs, 24 hs, 36 hs or 48 hs using a Nikon Eclipse TS100 microscope. For the invasion assay, the chamber inserts were coated with 40ul Matrigel matrix (BD Bioscience) and dried for two hours at 37°C. Both for the migration and invasion assay, 2-5×10^4^ cells in 500ul FBS-free medium were inoculated into upper chambers with the addition of 700ul 10% FBS medium in lower chambers. After incubating for 36 hs, 48 hs or 72 hs, the cells that had migrated to the underside of the membrane were fixed with 4% PFA, stained with 0.5% crystal violet, and subsequently counted in 5 microscopic fields. All experiments were independently repeated in triplicate.

**Anoikis assay**

The anoikis assay was performed using established protocols. ^2^ In brief, 5×10^6^ cells were starved in 0.5% FBS for 24 hs and counted and then suspended in 13 ml FBS-free medium in 15ml-Falcon tube. The cells were allowed to rock at 37°C for 24 hs and then pelleted and counted. The two cell counts were measured in triplicate and the ratio of the two average values were used to determine the percent viability.

**Mitochondrial mass determination**

The mitochondrial content was determined by the fluorescent intensity of MitoTracker®Red CM-H2 XRos (Invitrogen, M7513) as previously described ^3^ with the following modification: the concentration of MitoTracker probe was 500 nM.

**Mitochondrial membrane potential measurements**

Cells were cultured in DMEM containing 200 nM TMRM (Molecular Probes) at 37°C for 30 min. Then the medium was substituted for 1 mL fresh culture medium, and cells were imaged with confocal microscopy. Relative fluorescence was quantified using Image J.

**Extracellular Flux Analysis**

For oxygen consumption, 6×10^4^ cells were plated, and 16 hs later, oxygen consumption rate (OCR) was measured with the MitoXpress^®^Xtra (Luxcel Biosciences) under basal conditions or after addition of 5 μM FCCP (Sigma) and monitored as a function of fluorescence (excitation/emission 380/650nm) every 3 min over 100min on a time-resolved fluorescence (TRF) microplate reader (Tecan, Infinite M1000). Data were analyzed as described previously. ^4^

For excellular acid, 8×10^4^ cells per well were inoculated 16–18 hs prior to examination. excellular acid rate (ECAR) was measured with the pH-Xtra ^TM^ (Luxcel Biosciences) and monitored as a function of fluorescence (excitation/emission 380/610nm) every 3 min over 180min on a TRF microplate reader (Tecan, Infinite M1000). Data were analyzed using the equation described previously. ^5^

**ATP measurements**

ATP concentrations were quantified with an ATP Determination Kit (Molecular Probes) on a VarioSkan flash fluorescence plate reader (thermo scientific) according to the manufacturer’s instructions. The protein levels were determined with a BCA Protein Assay Kit (Beyotime, China) and were used for normalization. The ATP level was expressed as units of nmol per mg protein.

**Cellular ROS and GSH Levels**

For intracellular ROS levels, cells were incubated in the medium containing 10 μM dihydroethidium (DHE) (Molecular Probes) for 30min at 37°C in the dark. The medium was switched to fresh medium before fluorescent detection. The cellular glutathione levels were measured with a glutathione colorimetric detection kit (BioVision Research Products) according to the manufacturer's instructions.

**Glucose Consumption and Lactate Secretion**

Cells were seeded into culture plates and incubated for 5hs. The culture medium was then changed and cells were cultured for 16hs. The levels of glucose in the culture medium were measured using an assay kit from Nanjing Jiancheng Bioengineering Institute (Nanjing, China) following the manufacturer's recommendations. Lactate concentration was measured with a Lactate Assay Kit (Biovision Inc.) according to the manufacturer's instructions. Cells from each well were harvested and counted. The glucose consumption and lactate secretion were normalized to the cells number.

**Transmission Electron Microscopy**

The cells were scraped off, pelleted at 2,000 g for 5 min and resuspended in PBS. The cells were then pelleted again at 1,500 g for 5 min, fixed on ice for 4 h in 2.5% glutaraldehyde and postfixed in 1% osmium tetroxide buffer. The samples were then embedded in spur resin. Thin sections were cut, stained with a saturated solution of uranylacetate and lead citrate and observed at 80 kV using a JEOL 1200EX transmission electron microscope.

**siRNA transfection**

Silencing of PDHA1 and MPC1 was performed using 50 nM Dharmacon SmartPool siRNA [PDHA1 siRNA cat#: L-010329-00-0005; MPC1 siRNA cat# L-020459-01-0005]. siCon was a previously used scrambled siRNA sequence: GAG ACC CUA UCC GUG AUU A. ^6^ siRNA was introduced via Lipofectamine™ 2000 transfection reagent (Invitrogen) following the manufacturer’s protocols. After 48hs, qRT-PCR to determine the silencing efficiency and other subsequent experiments were performed.

**Statistical analysis**

Statistical analyses were performed using Graphpad Prism 5 software. Student's t-test or one-way ANOVA was applied to determine the significance between groups. Statistical analyses between different cell cohorts at different time points were performed using two-way ANOVA with Bonferroni's correction. The overall survival was estimated according to the Kaplan-Meier method, and significance was determined by the log-rank test. The statistical correlation between the clinical parameters of CCA patients and different PGC1α expression levels in Supplementary Table 1 was analyzed by chi-square test or Fisher’s exact chi-square test. The correlation between the expression of PGC1α and that of PDHA1 and MPC1 was analyzed by the Pearson correlation coefficient. Statistical significance was concluded at *P < 0.05, **P < 0.01, ***P < 0.001; n.s. represents no statistical significance.

**References**

1. Wang J, et al. N-myc downstream-regulated gene 2 inhibits human cholangiocarcinoma progression and is regulated by leukemia inhibitory factor/MicroRNA-181c negative feedback pathway. Hepatology. 2016;64:1606-1622. doi:10.1002/hep.28781.

2. LeBleu VS, et al. PGC-1α mediates mitochondrial biogenesis and oxidative phosphorylation to promote metastasis. Nat Cell Biol. 2014;16:992-1003. doi:10.1038/ncb3039.

3. Li Y, et al. SIRT1 facilitates hepatocellular carcinoma metastasis by promoting PGC-1α-mediated mitochondrial biogenesis. Oncotarget. 2016;7: 29255-29274. doi:10.18632/oncotarget.8711.

4. Newington JT, Rappon T, Albers S, Wong DY, Rylett RJ, Cumming RC. Overexpression of pyruvate dehydrogenase kinase 1 and lactate dehydrogenase A in nerve cells confers resistance to amyloid beta and other toxins by decreasing mitochondrial respiration and reactive oxygen species production. J Biol Chem. 2012;287:37245-37258. doi:10.1074/jbc.M112.366195.

5. Hynes J, Natoli EJ, Will Y. Fluorescent pH and Oxygen Probes of the Assessment of Mitochondrial Toxicity in Isolated Mitochondria and Whole Cells. Curr Protoc Toxicol. 2009;Chapter 2: Unit 2.16. doi:10.1002/0471140856.tx0216s40.

6. Shannon CE, Daniele G, Galindo C, Abdul-Ghani MA, DeFronzo RA, Norton L. Pioglitazone inhibits mitochondrial pyruvate metabolism and glucose production in hepatocytes. FEBS J. 2017;284:451-465. doi:10.1111/febs.13992.
